# CLOCK 3111T/C Polymorphism and Sex Moderate the Effect of Childhood Trauma on White Matter Microstructure in Bipolar Disorder

**DOI:** 10.3390/genes17080912

**Published:** 2026-07-31

**Authors:** Federica Nozza, Beatrice Bravi, Lidia Fortaner-Uyà, Alessia Giovanelli, Marco Paolini, Cristina Lorenzi, Sara Spadini, Greta D’Orsi, Cristina Colombo, Sara Poletti, Francesco Benedetti

**Affiliations:** 1 Psychiatry & Clinical Psychobiology, IRCCS Ospedale San Raffaele, 20132 Milano, Italy; bravi.beatrice@hsr.it (B.B.); fortaner.uyalidia@hsr.it (L.F.-U.); paolini.marco@hsr.it (M.P.); lorenzi.cristina@hsr.it (C.L.); spadini.sara@hsr.it (S.S.); dorsi.greta@hsr.it (G.D.); colombo.cristina@hsr.it (C.C.); poletti.sara@hsr.it (S.P.); benedetti.francesco@hsr.it (F.B.); 2 Faculty of Psychology, Vita-Salute San Raffaele University, 20132 Milan, Italy; a.giovanelli1@studenti.unisr.it

**Keywords:** clock genes, bipolar disorder, adverse childhood experiences, white matter integrity, sex

## Abstract

Background. Bipolar disorder (BD) is characterized by circadian rhythm disruptions, contributing to mood instability and recurrence. These rhythms are regulated by clock genes in the suprachiasmatic nucleus, including the CLOCK 3111T/C (rs1801260) polymorphism that has been linked to delayed sleep phase, insomnia, and altered circadian expression. Both circadian disruption and adverse childhood experiences (ACEs) correlate with white matter (WM) abnormalities. We hypothesized that rs1801260 moderates ACE effects on WM microstructure in BD. Methods. We enrolled 137 BD patients in depressive episodes. Participants underwent 3T MRI, rs1801260 genotyping and completed the Childhood Trauma Questionnaire. Moderation (PROCESS) tested genotype–ACE interactions on whole-brain fractional anisotropy (FA), axial diffusivity (AD), mean diffusivity (MD), and radial diffusivity (RD) values; voxel-wise TBSS (FSL Randomize) localized effects, with sex-stratified and GLZ analyses for genotype–sex interactions. Results. Significant rs1801260 × ACE interactions emerged for FA and RD across physical abuse, physical neglect, and emotional neglect. Higher ACEs were associated with lower FA/higher RD only in CLOCK rs1801260*C carriers, mainly females. TBSS showed physical abuse × rs1801260 interaction in the corpus callosum, internal capsule and corona radiata. A GLZ model with separate slopes confirmed physical abuse × sex × rs1801260 interactions on FA/RD, with effects specific to female CLOCK rs1801260*C carriers but genotype-independent in males. Conclusions. rs1801260 moderates the impact of early-life stress on WM integrity in BD, particularly in emotion-regulation tracts, with CLOCK rs1801260*C carriers showing greater vulnerability. Effects are genotype-specific in females but genotype-independent in males, possibly reflecting sex-dimorphic neurodevelopment driven by estrogen–androgen modulation of clock genes, HPA axis, and myelination.

## 1. Introduction

Bipolar disorder (BD) is a chronic psychiatric illness characterized by recurrent episodes of mania/hypomania and depression [1]. Increasing evidence suggests that circadian rhythm disruption represents a core pathophysiological feature of BD [2]. A shorter circadian phase and a phase delay in the sleep–wake cycle are accompanied by alterations in the timing and amount of cortisol and melatonin secretion with higher cortisol and lower melatonin levels [3]. In individuals with BD, irregularities in circadian timing are thought to contribute to mood instability and the recurrence of manic and depressive episodes [4].

The circadian system is composed of endogenous oscillators generating approximately 24 h cycles that govern fundamental physiological processes, including the sleep–wake rhythm, hormonal secretion, metabolism, neuronal activity, and immune responses [5,6,7]. The central pacemaker in the suprachiasmatic nucleus (SCN), mainly entrained by light signals, coordinates peripheral clocks through neural and hormonal pathways to ensure temporal coherence [6,7,8].

At the molecular level, circadian rhythms emerge from transcriptional–translational feedback loops of core clock genes (CLOCK, BMAL1, PER1-3, and CRY1-2), broadly expressed in the brain and involved in regulating neuronal excitability and affective behavior [9,10,11,12]. Circadian gene variations contribute to BD pathophysiology through effects on neurobiological pathways, including inflammation and neural activity regulation, and neurostructural alterations [13,14,15,16,17,18]. Several genetic association studies suggested associations between BD and some polymorphisms of CLOCK, BMAL1, and PER3 [3].

Among these variants, the CLOCK 3111T/C polymorphism (rs1801260, thymine [T] to cytosine [C] nucleotide substitution in the 3′-untranslated region of the CLOCK gene) influences the expression, function, and stability of CLOCK mRNA [17]. In humans, the *C variant has been associated with a slightly delayed circadian preference for activities in healthy subjects and with delayed sleep, insomnia, evening chronotype, and higher mood episode recurrence in patients with BD [19,20,21]. rs1801260 also influenced the neuropsychological performance and BOLD neural response to a moral valence decision task in BD patients during depression, confirming that it affects brain function [22].

Neuroimaging studies indicate that BD and chronic circadian disruption are associated with white matter (WM) microstructural alterations. Diffusion tensor imaging (DTI) studies in BD reveal altered WM microstructures with reduced fractional anisotropy (FA) and increased radial diffusivity (RD) in several tracts including the corpus callosum and cingulum, indicative of myelin impairment or axonal disorganization [23,24,25,26] in fronto-limbic and interhemispheric circuits implicated in emotional regulation and impulsivity control. Notably, CLOCK rs1801260*C carriers exhibit widespread MD increases, suggesting diffuse microstructural disorganization. Such alterations may represent one anatomical pathway through which genetic vulnerability translates into mood instability [27].

Finally, early-life stress acts as an environmental amplifier of this vulnerability. A history of childhood maltreatment is highly prevalent in patients with BD and has been associated with an earlier onset of the disorder, worse clinical course, and higher lifetime suicidal ideation and suicide attempts [28]. Adverse childhood experiences (ACEs) and circadian rhythms influence each other, with the circadian clock affecting sensitivity to stress and the HPA response system, while prolonged stress acts on the release of glucocorticoids and disrupts circadian gene expression both in peripheral and nervous tissues [29]. The interplay of ACE and alterations of circadian rhythms and their effect on the HPA can disrupt WM integrity and exacerbate illness onset, symptom severity, and susceptibility to autoimmune dysfunction [30]. ACEs have been associated with alterations in WM microstructure in patients with BD, particularly involving decreased axonal integrity in major tracts such as the corona radiata, corpus callosum, and uncinate fasciculus—pathways crucial for emotional and cognitive processing—thus highlighting a convergence between early-life stress, circadian dysregulation, and impaired structural connectivity [28]. A previous study from our group showed an interaction between ACE and the CLOCK 3111T/C polymorphism on the history of suicide [31], suggesting that CLOCK variants can moderate the effects of environmental stimuli on core features of BD.

These considerations led us to hypothesize that the CLOCK 3111T/C polymorphism could moderate the effect of ACE on WM microstructural integrity in core tracts for BD symptomatology. Here, we tested this hypothesis in a homogeneous sample of patients with BD.

## 2. Materials and Methods

### 2.1. Sample and Study Design

We studied 137 patients with BD consecutively admitted to the Mood Disorder ward at San Raffaele Turro hospital during an ongoing depressive episode. Patients had been referred for hospital specialized clinical treatment of depression by their general practitioners or psychiatrists in charge. Diagnosis was confirmed by staff psychiatrists according to DSM-5 criteria. Exclusion criteria were current diagnosis of any additional psychiatric disorder, including alcohol and/or substance dependence or abuse in the last 6 months, intellectual disability, pregnancy, and major medical and neurological disorders.

All patients underwent 3 T Magnetic Resonance scanning, venous blood sampling for genotyping, and completed the Childhood Trauma Questionnaire (CTQ). For all patients, lithium treatment was recorded. In addition, 107 healthy controls (HC), matched with patients with BD for age and sex (Table S1), underwent 3 T Magnetic Resonance scanning; however, genotyping and Childhood Trauma Questionnaire (CTQ) data were not available for these subjects.

After a complete description of the study, written informed consent was obtained. All research activities have been approved by the local Ethical Committee.

### 2.2. Assessment of Adverse Childhood Experiences

Childhood maltreatment was assessed using the 28-item Childhood Trauma Questionnaire (CTQ) [32], a psychometric scale with good internal reliability (Cronbach’s α = 0.93–0.99) [33]. The CTQ includes five subscales (physical abuse, emotional abuse, sexual abuse, physical neglect, and emotional neglect), that we summed to also obtain a total score.

Both total score and subscales were used in the statistical and neuroimaging analyses as continuous variables to test their effects on DTI measures. All the neuroimaging and statistical analyses were corrected for a minimization score, which was computed as in Bernstein et al., 2003 [32]. Higher scores indicate a greater tendency to minimize or deny experiences of childhood maltreatment, suggesting possible underreporting of adversity.

### 2.3. Genotype Data Processing and Quality Control

Venous blood samples were collected from all participants to obtain genetic data. For 112 patients, genotyping was performed using the Infinium PsychArray-24 BeadChip (Illumina, Inc., San Diego, CA, USA). This high-density microarray, developed in collaboration with the Psychiatric Genomics Consortium, features 595,427 markers specifically selected for their association with psychiatric conditions. An additional 25 patients were genotyped using the Infinium Global Screening Array-24 Kit (Illumina, Inc., San Diego) supplemented with the Psych Booster, encompassing 654,027 fixed markers along with 30,000 variants derived from the Infinium PsychArray-24 BeadChip.

Looking at genetic variants mapping to the CLOCK gene region, the genetic variant rs1801260 was found to be included in both arrays. Before extracting its genotype data, genotypic data processing and quality control were executed using PLINK v1.9 [34]. First, we excluded genetic markers characterized by a minor allele frequency (MAF) of less than 5%, a call rate below 95%, or a significant deviation from Hardy–Weinberg equilibrium at *p* < 10^−6^. Subsequently, individual samples were removed if they exhibited discrepancies between genotypic and phenotypic sex, a total genotype call rate of less than 95%, or outlying autosomal heterozygosity defined as Fhet > ±0.2. In addition, we accounted for the relatedness of participants by excluding one member of any participant pair with an identity-by-descent (IBD) exceeding 0.1875, which is the threshold for the halfway between third- and second-degree relatives [35]. Finally, European population ancestry was ensured by removing individuals that exceeded 5 standard deviations from the mean of the first two genetic principal components.

### 2.4. Image Acquisition and Preprocessing

Both patients and healthy controls underwent 3 Tesla MRI acquisition at C.E.R.M.A.C. (Centro d’Eccellenza di Risonanza Magnetica ad Alto Campo) in San Raffaele Hospital in Milan. A total of 64 patients and 52 healthy controls were scanned with Philips Gyroscan Intera (Philips, Best, The Netherlands); 73 patients and 55 healthy controls were scanned with Philips Ingenia CX scanner (Philips, Best, The Netherlands). DWI was executed using SE Eco-planar imaging and the following parameters for the first scanner: TR/TE = 8753.89/58 ms, FoV (mm) 231.43 (ap), 126.50 [36], 240.00 (rl); acquisition matrix 2.14 × 2.71 × 2.31; 55 contiguous, 2.3 mm thick axial slices reconstructed with in-plane pixel size 1.88 × 1.87 mm; SENSE acceleration factor = 2; 1 b0 and 35 non-collinear directions of the diffusion gradients; b value = 900 s/mm2. The following parameters were for the second Philips scanner: TR/TE = 5900/78 ms, FoV (mm) 240 (ap), 129 (5), 232 (rl); acquisition matrix 2.14 × 2.73 × 2.30; 56 contiguous, 2.3 mm thick axial slices reconstructed with in-plane pixel size 1.88 × 1.88 × 2.30 mm; SENSE acceleration factor = 2; 1 b0 and 40 non-collinear directions of the diffusion gradients; b value = 1000 s/mm^2^. Fat saturation was performed to avoid chemical shift artifacts. DTI analysis and tensor calculations were carried out using the “Oxford Center for Functional Magnetic Resonance Imaging of the Brain Software Library” (FSL 6.0; www.fmrib.ox.ac.uk/fsl/index.html) [37]. First, removal of nonbrain tissue was performed with a brain extraction tool (BET) provided by FSL, supplying a brain mask for each subject. Then, the FSL eddy tool was used, which handles a Gaussian process to predict undistorted data to which the actual volumes can be aligned [38]. It can estimate and correct for volume-to-volume movement and off-resonance fields, signal dropout due to movement during the diffusion encoding, and intra-volume movement [39]. After, least-square fits were performed to estimate the fractional anisotropy (FA), eigenvector, and eigenvalue maps. Mean diffusivity (MD) was defined as the mean of all three eigenvalues (λ1 + λ2 + λ3)/3, axial diffusivity (AD) as the principal diffusion eigenvalue (λ1), and radial diffusivity (RD) as the mean of the second and third eigenvalues (λ2 + λ3)/2. Next, all individuals’ volumes were skeletonized and transformed into a common space as used in Tract-Based Spatial Statistics (TBSS) [37]. Briefly, all volumes were nonlinearly warped to the FMRIB58_FA template supplied with FSL (http://www.fmrib.ox.ac.uk/fsl/tbss/FMRIB58_FA.html) and normalized to the Montreal Neurological Institute space, by use of local deformation procedures performed by FMRIB’s Non-Linear Image Registration Tool (FNIRT). Next, a mean FA volume of all subjects was generated and thinned to create a mean FA skeleton representing the centers of all common tracts. We thresholded and binarized the mean skeleton at FA > 0.2 to reduce the likelihood of partial voluming in the borders between tissue classes. Individual FA values were warped onto this mean skeleton mask. The resulting tract invariant skeletons for each participant were fed into voxel-wise permutation-based cross-subject statistics. Similar warping and analyses were used on MD, AD, and RD data.

### 2.5. Statistical Analysis

To explore the interaction between rs1801260 polymorphism and ACEs on WM microstructure, whole-brain average FA, MD, RD, AD values were extracted for each subject. Because of the small number of C/C homozygotes, they were pooled together with the corresponding heterozygotes (T/C), also considering evidence from the literature suggesting shared clinical features; therefore, all analyses compared *C allele carriers with T/T homozygotes [14,19,27,31,40,41,42,43,44,45,46].

Preliminary moderation analyses were performed using the SPSS “Process” Macro (version 4.0) in IBM SPSS Statistics for Windows, Version 30.

All CTQ scores were entered as continuous independent variables, and whole-brain average FA, MD, RD, and AD values were entered as dependent variables. The rs1801260 polymorphism (*C carriers and T/T homozygotes) was entered as moderator. Age, sex, lithium treatment and CTQ minimization subscale were used as nuisance covariates. To account for the two different MRI scanners, a “scan” nuisance covariate was also added to all analyses. Moderation analyses were then repeated separately in male and female groups.

Then, voxel-wise DTI analyses were performed using nonparametric permutation-based testing as implemented in Randomise in FSL. All analyses were carried out in the context of the general linear model (GLM), and the same nuisance covariates used in moderations analyses were included in the model.

Firstly, we conducted a voxel-wise one-way ANOVA (TFCE-corrected, *p* < 0.05) across *C carriers, TT homozygotes, and healthy controls for each DTI metric (FA, MD, RD, AD)—using sex, age, and scan as covariates—to disentangle the specific contribution of the CLOCK rs1801260 polymorphism; then, we performed voxel-wise analyses to examine whether DTI metrics (FA, MD, RD, and AD) differed between rs1801260 groups (*C carriers vs. T/T homozygotes), using age, sex, lithium and scan as covariates.

To investigate whether the relationship between ACEs and WM differs between the two rs1801260 groups, we performed voxel-wise separate slopes analyses, entering CTQ subscale scores (physical abuse, physical neglect, emotional abuse, emotional neglect, and sexual abuse), separately as continuous independent variables. Group differences in DTI metrics were tested using two-sample *t*-tests. We then conducted separate voxel-wise regression analyses within each rs1801260 group, regressing ACE scores on WM measures to explore group-specific association patterns.

Threshold-free cluster enhancement (TFCE) was used to avoid defining arbitrary cluster-forming thresholds and smoothing levels. The data were tested against an empirical null distribution generated by 5000 permutations for each contrast, thus providing statistical maps fully corrected for multiple comparisons across space. Corrected *p* < 0.05 was considered significant.

To examine potential sex-specific effects, the full sequence of analyses was repeated separately in male and female subsamples.

Finally, participants were stratified by rs1801260 and sex, and group differences in mean FA and RD values were tested using a GLZ separate slope analyses.

## 3. Results

### 3.1. Clinical and Demographic Characteristics

Table 1 and Table 2 summarize the clinical and demographic characteristics of the sample by genotype and sex, respectively. The sample included 137 BD patients (76 T/T homozygotes and 61 *C carriers; 85 females and 52 males).

No significant differences emerged between genotype groups in demographic or clinical variables or CTQ total/subscale scores, except for emotional abuse, which was significantly higher in T/T homozygotes than in *C carriers (T/T:9.11 ± 4.62; *C: 7.49 ± 3.04; t(130.358) = 2.45, *p* = 0.015).

Similarly, no significant differences emerged between sex groups in demographic or clinical variables or CTQ total/subscale scores, except for sexual abuse, which was higher in females than in males (females: 5.55 ± 1.49; males: 5.14 ± 0.57; t(118.387) = 2.223, *p* = 0.028).

### 3.2. Moderation Analyses by rs1801260 on Mean Whole-Brain WM Metrics

rs1801260 significantly moderated the effect of exposure to childhood trauma on whole-brain FA and RD mean values. Effects were detected for several dimensions of childhood trauma and were significantly influenced by sex.

**Figure 1:**
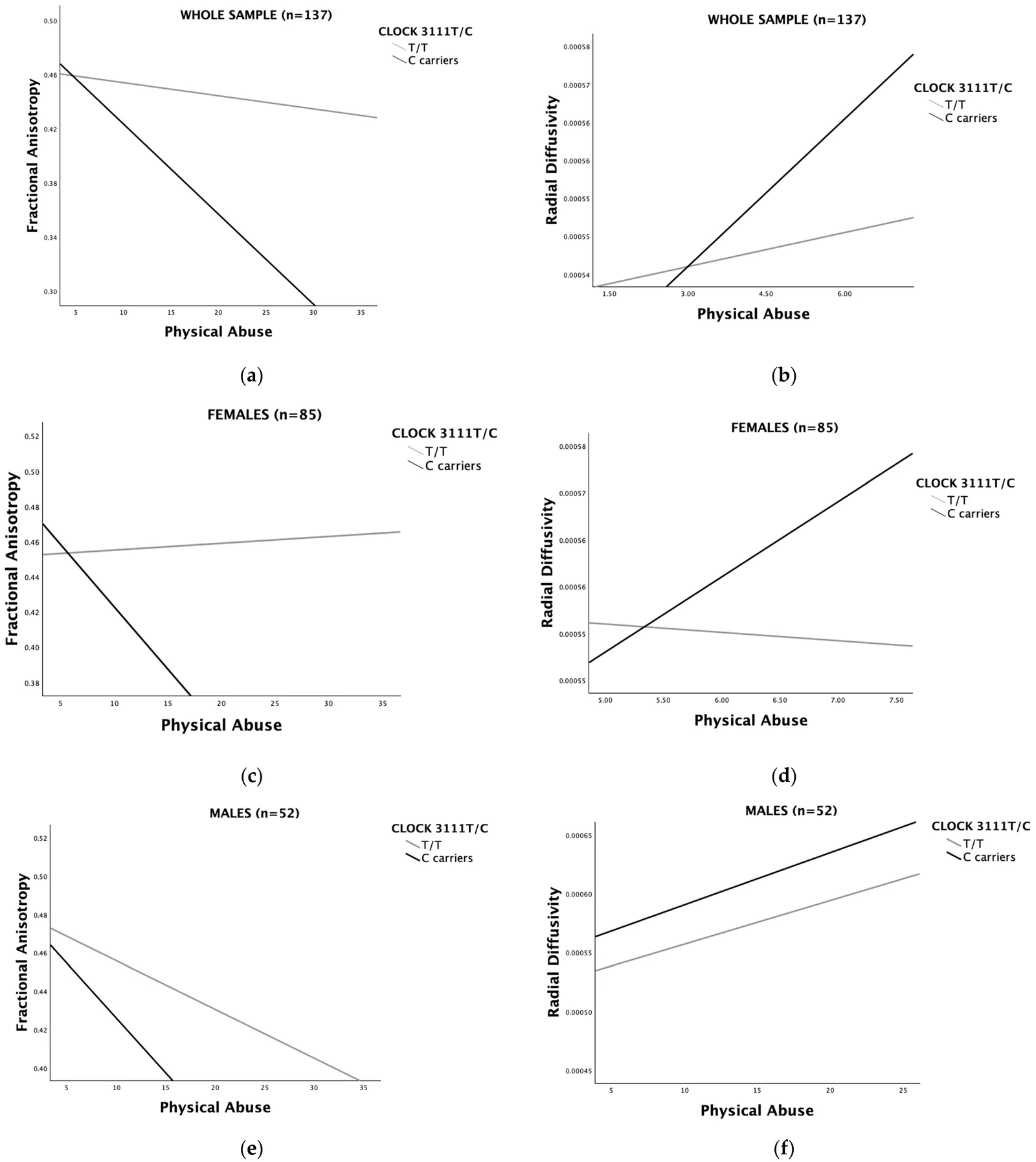
(**a**) Interaction between CTQ physical abuse and rs1801260 on FA in the whole sample. (**b**) Interaction between CTQ physical abuse and rs1801260 on RD in the whole sample; (**c**) interaction between CTQ physical abuse and rs1801260 on FA in females; (**d**) interaction between CTQ physical abuse and rs1801260 on RD in females; (**e**) interaction between CTQ physical abuse and rs1801260 on FA in males; (**f**): interaction between CTQ physical abuse and rs1801260 on RD in males.

Analyses revealed a significant interaction between physical abuse and rs1801260 in predicting mean FA (F (1128) = 8.76, *p* = 0.003) (Figure 1a) and a trend-level interaction for mean RD (F (1128) = 3.66, *p* = 0.057) (Figure 1b). Specifically, higher physical abuse scores were associated with decreased mean FA (b = −0.006, t = −4.067, *p* < 0.001) and increased mean RD (b = 0.000, t = 2.791, *p* = 0.006) in *C carriers, but not in T/T homozygotes.

Emotional neglect similarly interacted with rs1801260 to predict mean FA (F(1, 128) = 7.029, *p* = 0.009) and mean RD (F(1, 128) = 6.206, *p* = 0.014) (Supplementary Figure S1a,b). Among *C carriers, higher emotional neglect scores were associated with decreased mean FA (b = −0.001, t = −2.015, *p* = 0.046) and increased mean RD (b = 0.000, t = 2.189, *p* = 0.030), while no significant effect was found in T/T homozygotes.

Physical neglect also interacted with rs1801260 polymorphism to predict mean FA (F(1, 128) = 5.273, *p* = 0.023) and mean RD (F(1, 128) = 3.883, *p* = 0.050) (Supplementary Figure S2a,b). Higher physical neglect scores were associated with decreased mean FA (b = −0.002, t = −2.806, *p* = 0.005) and increased mean RD (b = 0.000, t = 2.131, *p* = 0.035) in *C carriers, but not in T/T homozygotes.

Sex-stratified analyses indicated that these effects emerged exclusively in female *C carriers but not in males (Figure 1e,f, Figures S1e,f and S2e,f). Specifically, in females, physical abuse and rs1801260 significantly interacted in predicting mean FA (F (1, 77) = 18.33, *p* < 0.001) (Figure 1c) and mean RD (F (1, 77) = 11.03, *p* = 0.001) (Figure 1d). Among female *C carriers, higher physical abuse scores were associated with lower mean FA (b = −0.007, t = −5.005, *p* < 0.001) and higher mean RD (b = 0.000, t = 4.009, *p* < 0.001), whereas no such associations were observed in T/T homozygotes.

Emotional neglect and rs1801260 significantly interacted in predicting mean FA (F (1, 77) = 9.11, *p* = 0.003) and mean RD (F(1, 77) = 7.71, *p* = 0.007) in females (Supplementary Figure S1c,d). Among female *C carriers, higher emotional neglect scores were associated with lower mean FA (b = −0.002, t = −2.365, *p* = 0.020) and higher mean RD (b = 0.000, t = 2.047, *p* = 0.040), while no significant effects were detected in T/T homozygotes.

Similarly, physical neglect and rs1801260 significantly interacted in predicting mean FA (F(1, 77) = 11.29, *p* = 0.001) and mean RD (F(1, 77) = 9.05, *p* = 0.004) in females (Supplementary Figure S2c,d). Among female *C carriers, higher emotional neglect scores were associated with lower mean FA (b = −0.004, t = −3.299, *p* = 0.001) and higher mean RD (b = 0.000, t = 2.160, *p* = 0.033), while no significant effects were detected in T/T homozygotes.

### 3.3. Moderation Analyses by rs1801260 on Voxel-Wise WM Analyses

Voxel-wise one-way ANOVA revealed a significant main effect of group on all diffusion metrics (FA, MD, RD, AD; TFCE-corrected, *p* < 0.05). Post hoc comparisons indicated that *C carriers differed from both TT homozygotes and healthy controls across multiple WM tracts, whereas no significant differences were observed between TT homozygotes and healthy controls. Detailed statistics and tract-wise localizations are provided in the Supplementary Materials (Supplementary Results, Figure S3a–d).

Subsequently, voxel-wise WM analyses (TFCE-corrected, *p* < 0.05) were conducted to assess differences in diffusion metrics (FA, MD, RD, AD) between *C carriers and TT homozygotes and between males and females. A significant main effect of rs1801260 was observed for AD (*p* = 0.020), RD (*p* = 0.017), and MD (*p* = 0.015) (Supplementary Figure S4a–c), with *C carriers exhibiting significantly higher values compared to T/T homozygotes across widespread white matter tracts, such as the corpus callosum, internal capsule, corona radiata. No statistically significant difference in FA was observed between CLOCK genotype groups (*p* = 0.088), although a trend toward higher FA in T/T homozygotes relative to *C carriers was observed.

Importantly, no significant sex differences were detected for any diffusion metric (FA, MD, RD, or AD), indicating that WM integrity measures did not differ between males and females in this sample.

**Figure 2:**
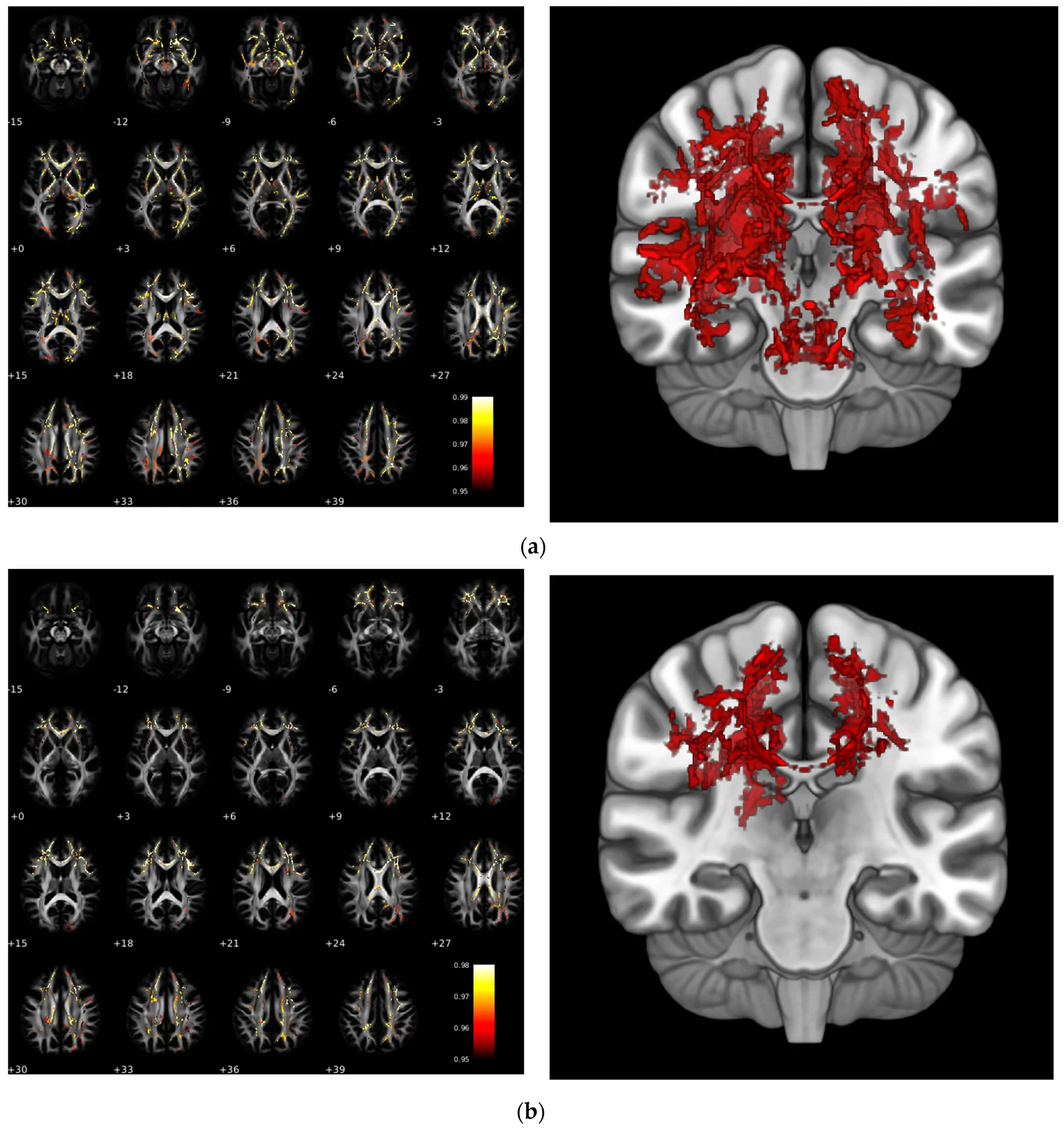

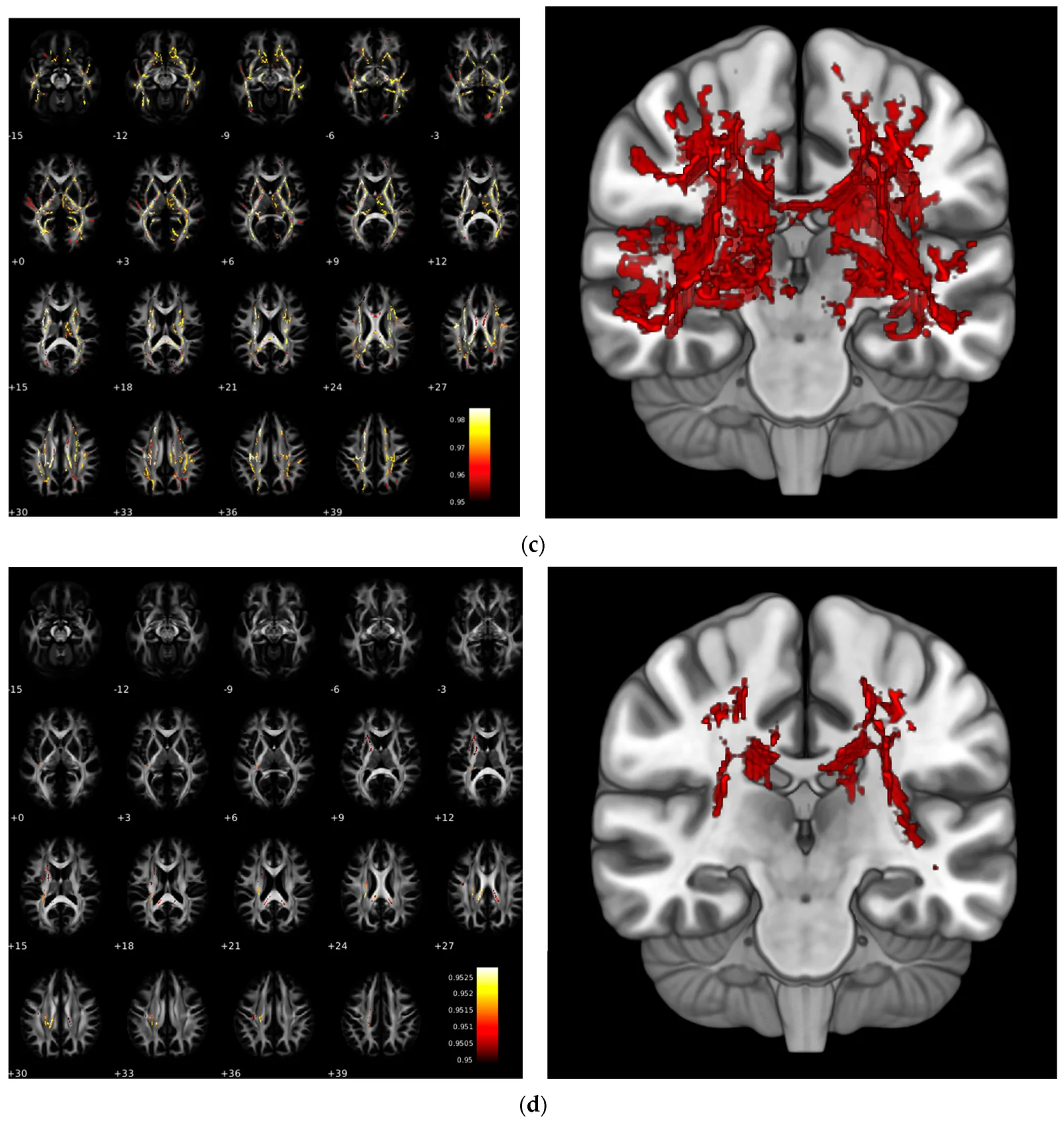
(**a**) White matter tracts where CTQ physical abuse is negatively associated with FA in *C carriers. The color bar refers to 1 − *p* values for the association at TFCE analysis. Numbers are z coordinates in the standard Montreal Neurological Institute (MNI) space. The panel on the right shows a coronal section of the same significant white matter tracts in 3D rendering; (**b**) white matter tracts where CTQ physical abuse is positively associated with RD in *C carriers. The color bar refers to 1 − *p* values for the association at TFCE analysis. Numbers are z coordinates in the standard Montreal Neurological Institute (MNI) space. The panel on the right shows a coronal section of the same significant white matter tracts in 3D rendering; (**c**) white matter tracts where CTQ physical abuse is negatively associated with FA in T/T homozygotes. The color bar refers to 1 − *p* values for the association at TFCE analysis. Numbers are z coordinates in the standard Montreal Neurological Institute (MNI) space. The panel on the right shows a coronal section of the same significant white matter tracts in 3D rendering; (**d**) white matter tracts where CTQ physical abuse is positively associated with RD in T/T homozygotes. The color bar refers to 1 − *p* values for the association at TFCE analysis. Numbers are z coordinates in the standard Montreal Neurological Institute (MNI) space. The panel on the right shows a coronal section of the same significant white matter tracts in 3D rendering. CTQ: Childhood Trauma Questionnaire; FA: fractional anisotropy; RD: radial diffusivity.

Voxel-wise TBSS analyses (TFCE-corrected, *p* < 0.05) testing the ACE-by-group interaction revealed no significant effects. Separate voxel-wise regression analyses conducted within each group showed that only physical abuse scores significantly affected WM microstructure, with decreased FA and increased RD values observed in both *C carriers (FA: *p* = 0.010; RD: *p* = 0.020) (Figure 2a,b and Figure S5a–d) and T/T homozygotes (FA: *p* = 0.016; RD: *p* = 0.048) (Figure 2c,d and Figure S6a–d), although for the latter the effect on RD was only marginally significant. In *C carriers, alterations in both FA and RD were observed in the genu, body, and splenium of the corpus callosum, the anterior, superior, and posterior corona radiata bilaterally, and the posterior thalamic radiation bilaterally. FA further involved the anterior and posterior limbs of the internal capsule bilaterally, while RD additionally showed involvement of the superior longitudinal fasciculus bilaterally. In T/T homozygotes, alterations in both FA and RD were detected in the body and splenium of the corpus callosum, the anterior limb of the internal capsule bilaterally, the posterior limb of the internal capsule on the left, the retrolenticular part of the internal capsule bilaterally, the anterior, superior, and posterior corona radiata, the posterior thalamic radiation on the left, and the superior longitudinal fasciculus on the left, with FA also involving the genu of the corpus callosum and the posterior thalamic radiation bilaterally (Table 3).

**Figure 3:**
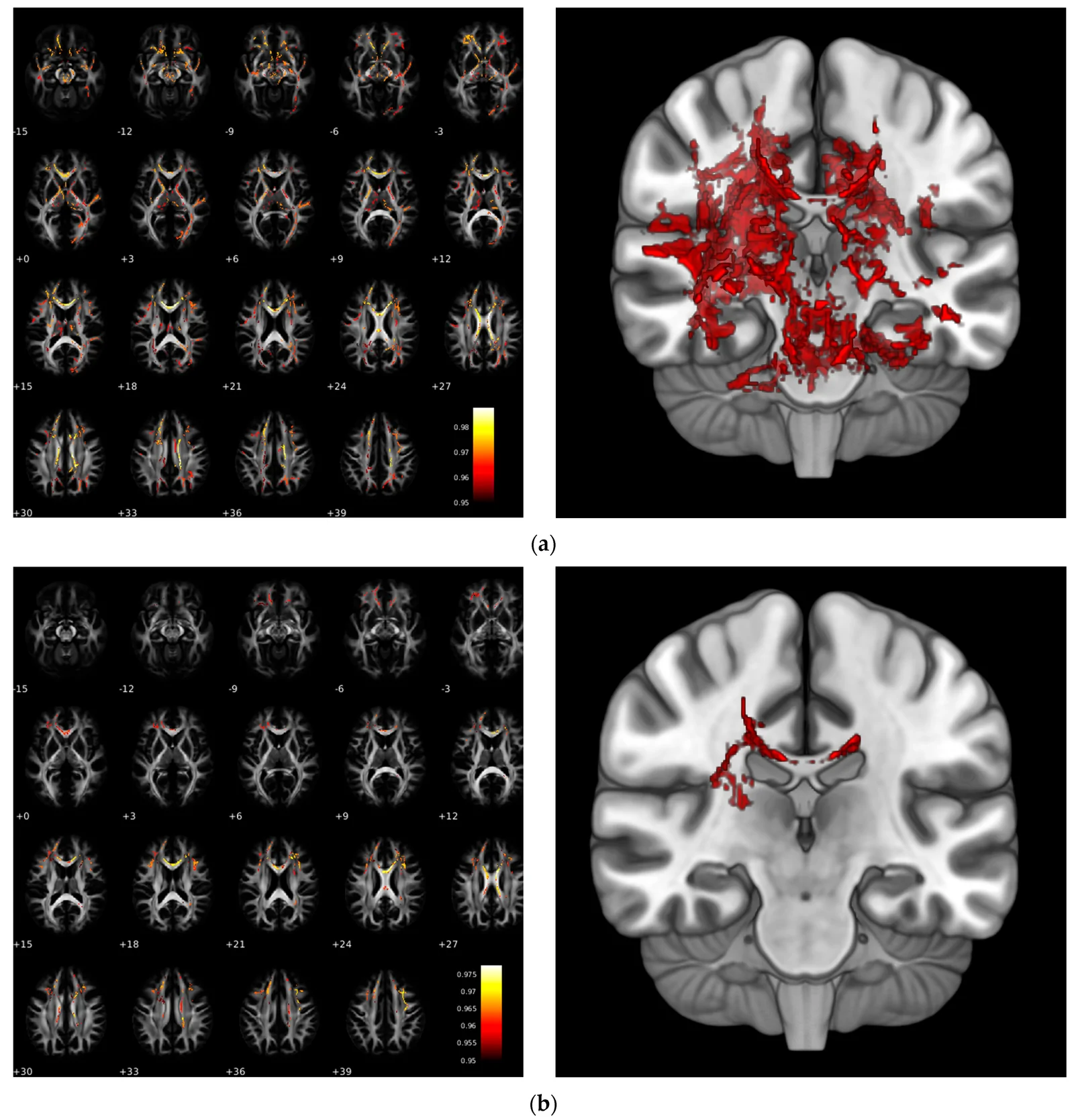

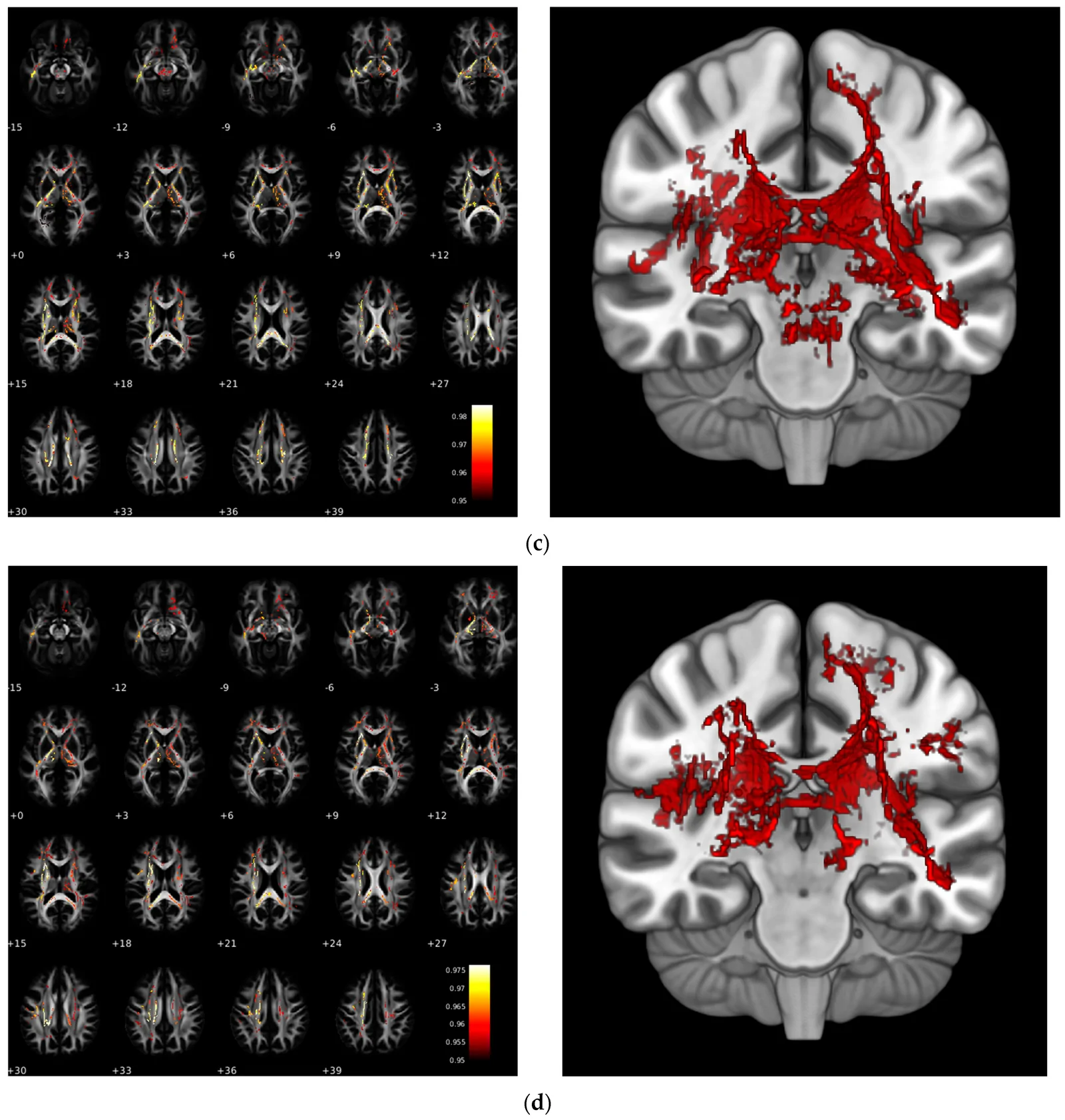
(**a**) White matter tracts where CTQ physical abuse is negatively associated with FA in females*C carriers. The color bar refers to 1 − *p* values for the association at TFCE analysis. Numbers are z coordinates in the standard Montreal Neurological Institute (MNI) space. The panel on the right shows a coronal section of the same significant white matter tracts in 3D rendering; (**b**) white matter tracts where CTQ physical abuse is positively associated with RD in females*C carriers. The color bar refers to 1 − *p* values for the association at TFCE analysis. Numbers are z coordinates in the standard Montreal Neurological Institute (MNI) space. The panel on the right shows a coronal section of the same significant white matter tracts in 3D rendering. (**c**) White matter tracts where CTQ physical abuse is negatively associated with FA in males T/T homozygotes. The color bar refers to 1 − *p* values for the association at TFCE analysis. Numbers are z coordinates in the standard Montreal Neurological Institute (MNI) space. The panel on the right shows a coronal section of the same significant white matter tracts in 3D rendering; (**d**) white matter tracts where CTQ physical abuse is positively associated with RD in males T/T homozygotes. The color bar refers to 1 − *p* values for the association at TFCE analysis. Numbers are z coordinates in the standard Montreal Neurological Institute (MNI) space. The panel on the right shows a coronal section of the same significant white matter tracts in 3D rendering; FA: fractional anisotropy. RD: radial diffusivity. CTQ: Childhood Trauma Questionnaire.

Sex-stratified voxel-wise TBSS analyses (TFCE-corrected, *p* < 0.05) testing the ACE-by-group interaction revealed no significant effects. Separate voxel-wise regression analyses conducted within each group indicated that physical abuse was significantly associated with decreased FA and increased RD values in female *C carriers (FA: *p* = 0.013; RD: *p* = 0.023) (Figure 3a,b and Figure S7a–d), whereas in males the effect was specifically driven by T/T homozygotes (FA: *p* = 0.016; RD: *p* = 0.024) (Figure 3c,d and Figure S8a–d). In female CLOCK*C carriers, alterations in both FA and RD were observed in the genu, body, and splenium of the corpus callosum, the anterior and superior corona radiata bilaterally, and the posterior corona radiata, with FA also involving the anterior and posterior limbs of the internal capsule bilaterally and the posterior thalamic radiation bilaterally, while RD additionally showed involvement of the posterior corona radiata in the right hemisphere. In male T/T homozygotes, alterations in both FA and RD were found in the genu, body, and splenium of the corpus callosum, the anterior limb of the internal capsule bilaterally, the posterior limb of the internal capsule bilaterally, the retrolenticular part of the internal capsule bilaterally, the anterior, superior, and posterior corona radiata bilaterally, and the posterior thalamic radiation bilaterally, with FA further extending to the superior longitudinal fasciculus (Table 4).

### 3.4. Physical Abuse Effects on FA and RD by Sex and CLOCK Genotype

A GLZ model with separate slopes significantly predicted both FA [LR χ^2^(4) = 31.54, *p* < 0.001] and RD [LR χ^2^(4) = 103.59, *p* < 0.001]. In both cases, the physical abuse × sex × rs1801260 interaction was significant [Wald χ^2^(4) = 12.77, *p* = 0.012 for FA; Wald χ^2^(4) = 14.35, *p* = 0.006 for RD], indicating that physical abuse was differentially associated with WM microstructure across sex and rs1801260 combinations.

In particular, analyses revealed significant inverse associations between physical abuse and WM microstructure in female *C carriers (*p* = 0.012 for FA; *p* = 0.018 for RD) but regardless of genotype in males (*p* *C = 0.013, *p* T/T = 0.044 for FA; *p* *C = 0.003, *p* T/T = 0.013 for RD) (Table 5 and Table 6).

## 4. Discussion

This study provides novel evidence that the CLOCK 3111T/C polymorphism (rs1801260) moderates the association between early-life stress and WM microstructure in BD and that this effect is sex-dependent.

Specifically, rs1801260*C female carriers who experienced childhood trauma (particularly physical abuse, physical neglect, and emotional neglect) exhibited whole-brain lower FA and higher RD mean values. Voxel-wise analyses confirmed these associations, especially for physical abuse, where both *C carriers and T/T homozygotes showed reduced FA and elevated RD across several WM tracts. Sex-specific analyses showed that the effect was present in female *C carriers and male TT homozygotes in the same tracts.

The moderating role of CLOCK gene on the effect of ACE in depression is in line with a previous study showing that CLOCK contributes to depressive symptoms by mediating the effects of early adversities and recent stressors [47].

These results can be interpreted as the combined effect of multiple, non-alternative pathogenetic mechanisms. The 3111T/C polymorphism of the CLOCK gene has been linked to the regulation of the sleep–wake rhythm and chronotype preferences [48,49,50,51]. In particular, *C carriers tend to display a stronger inclination toward eveningness, characterized by delayed sleep onset and increased daytime sleepiness [52]. Clock genes also contribute to the regulation of cellular homeostasis and oligodendrocyte maturation [27]. Circadian misalignment, such as phase delay and decreased amplitude of the rhythms, has been associated with reduced myelin density and reduced expression of myelin basic proteins, respectively in the corpus callosum and in the hippocampus [53]. Further, prolonged exposure to stress may induce reduction of the myelination index and expression of myelin basic proteins. Similarly, in glial cultures, stress exposure caused shrinkage and simplification in myelin basic proteins and oligodendrocyte processes [54]. Disruptions in circadian rhythms or clock gene function, due to early stress, can impair oligodendrocyte maturation and myelination, potentially contributing to microstructural abnormalities observable through DTI measures. In this framework, the *C allele might confer increased biological sensitivity to environmental stressors, translating circadian and sleep disturbances into altered white matter maturation [27,51]. Indeed, sleep promotes myelination and oligodendrocyte precursor cell proliferation, and it is associated with increased expression of genes involved in myelination within oligodendrocytes. Therefore, disruption of the sleep–wake cycle may impair these processes [55].

Another possible explanation for the joint effect of the CLOCK 3111T/C polymorphism and ACE on white matter microstructure involves their potential impact on neurotransmitter systems. Most neurotransmitters, including dopamine, serotonin, noradrenaline, glutamate and GABA, exhibit circadian fluctuations [56,57,58,59] and ACE has been shown to induce long-lasting reductions in serotonergic function [60]. Proliferation, differentiation, and myelination processes of oligodendrocytes are in part controlled by neurotransmitters [61]; therefore, factors affecting neurotransmission, such as clock gene variants and ACE, could influence oligodendrocytes cell fate.

Higher RD values may reflect loss of membrane integrity or alterations in fiber coherence, while decreased FA indicates poorer fiber organization, lower directional coherence, and/or reduced fiber integrity. These findings point to microstructural alterations indicative of reduced fiber coherence and potential demyelination, processes long implicated in the pathophysiology of BD [27,62,63,64,65].

The involvement of tracts such as the corpus callosum and corona radiata is particularly relevant given their roles in interhemispheric integration, emotion regulation, and cognitive control—domains frequently disrupted in bipolar disorder [66,67,68].

The corpus callosum is among the most consistently implicated structures in the DTI literature on BD; reduced integrity in this tract has been linked to impaired interhemispheric integration, attention, and contextual processing [23,69], while reduced integrity in the corona radiata has been consistently linked to impulsivity and emotional dysregulation [69]. Moreover, we found alterations in the internal capsule, thalamic radiations, and superior longitudinal fasciculus which are also frequently compromised in BD [70,71]. In particular, the internal capsule, especially its anterior limb, supports prefrontal, thalamic, and brainstem connectivity and is implicated in emotion, motivation, cognition, and decision-making [72]. Similarly, the thalamic radiations are implicated in the communication between cortical and subcortical systems, while the superior longitudinal fasciculus links frontal, parietal, and temporal regions involved in cognitive control and emotional regulation [73].

The association with childhood stress is also supported by the broader literature on early life adversity. A systematic review reported lower FA in tracts including the anterior thalamic radiation, corona radiata, cingulum, uncinate fasciculus, and inferior fronto-occipital fasciculus following early adversity [74]. Similarly, another study found that childhood trauma was associated with lower FA in the bilateral internal capsule, suggesting that early stress may leave long-lasting microstructural effects on pathways supporting sensory integration and limbic connectivity [75]. Overall, these data suggest that the interaction between circadian genetic vulnerability and adverse childhood experiences may contribute to abnormalities in the corpus callosum, corona radiata, internal capsule, thalamic radiations, and associative fibers, thereby promoting affective instability and cognitive-emotional dysfunction [66,68,69,76,77,78].

The presence of sex-specific effects may reflect divergent neurodevelopmental trajectories shaped by sex hormones, chromosomes, genotypes, and environmental exposures [72]. Although a well-known dimorphism is present in WM in healthy controls, where males typically exhibit higher FA and lower RD than females, driven primarily by androgen/estrogen exposures during sensitive developmental windows [73,74,75], here we observed no difference, in agreement with the literature in BD which is more controversial with studies showing both higher, lower, and similar WM in males and females [79,80].

The traditional framework of brain sexual differentiation highlights the central influence of steroid hormones. According to this view, exposure to elevated androgen levels during the prenatal and/or early postnatal stages of neural development exerts a lasting, or “organizational,” effect on the brain [73]. Such differences likely arise from sexually divergent neurobiological mechanisms, notably pubertal hormones [73] and gene–environment interactions [72,76,77], including sex-specific timing of clock gene expression (earlier in females) [78] and estrogen modulation of circadian rhythms [81]. In females, heightened vulnerability in *C-allele carriers aligns with estrogen facilitation of clock gene expression and HPA axis hyperreactivity to early stress, amplifying trauma’s demyelinating impact during accelerated myelination [78,81,82,83]. Importantly, the emergence of sex differences in HPA axis reactivity and sensitivity coincides with the increasing influence of gonadal hormones. Such variations in stress responsiveness have been associated with the higher prevalence of affective and anxiety disorders observed in females [83]. Males, conversely, demonstrate resilience through androgen-promoted axonal integrity and prolonged maturation, rendering WM alterations broadly trauma-responsive irrespective of CLOCK status [75,76,77,78,81,82,83,84,85]. By influencing the efficiency of neural signal transmission across brain regions, WM abnormalities may contribute to the dysregulated emotional responses, cognitive impairments, and behavioral alterations that characterize psychiatric disorders [86,87].

These results suggest that the rs1801260 may act as a genetic vulnerability factor that amplifies the neural consequences of childhood maltreatment, promoting WM alterations that are known to contribute to mood instability in BD [88].

These findings should be interpreted considering some limitations. Recruitment was conducted at a single center and within a single ethnic group, which may limit the generalizability of our findings due to potential population stratification. A key limitation of this study is that genotyping data were not available for the healthy control group, precluding direct comparisons between genetically defined bipolar disorder subgroups and equivalently stratified healthy individuals. Moreover, the cross-sectional design precludes causal inferences about the observed associations. As is common in studies of early adversity, ACEs were assessed retrospectively; while such measures are inherently sensitive to recall and reporting biases, we attempted to mitigate this by including the CTQ minimization subscale as a nuisance covariate. Participants were not drug-naïve, and pharmacological treatments administered over the course of illness may have influenced DTI metrics. To partially control for this, lithium exposure was included as a nuisance covariate; however, given the naturalistic design of our study, it was not possible to account for the full heterogeneity of pharmacotherapies.

Finally, conventional DTI metrics provide indirect markers of WM microstructure and do not allow direct inference regarding the underlying tissue geometry or time-dependent diffusion processes. Advanced theoretical frameworks and biophysical models of diffusion MRI have been proposed to better characterize tissue microstructure and may provide further mechanistic insights in future studies [89].

## 5. Conclusions

To the best of our knowledge, this is the first study to examine the moderating role of a CLOCK gene polymorphism in the well-established relationship between ACEs and WM alterations in BD. This represents a novel contribution to the understanding of gene–environment interactions underlying neurobiological vulnerability in mood disorders. Future longitudinal studies will be essential to confirm these findings and to clarify why individuals carrying CLOCK gene variants may show increased susceptibility to the neurobiological effects of early-life trauma. Moreover, sex-stratified analyses allowed us to detect a sexually dimorphic modulation of the rs1801260 x childhood trauma interplay on WM microstructure, in line with emerging evidence of sex differences in WM integrity and stress- and clock-related mechanisms in mood disorders. By explicitly modeling sex effects, our findings contribute to clarifying how sex-specific neurodevelopmental trajectories and hormonal differences shape vulnerability to circadian and white matter alterations in bipolar disorder.

These results further underscore the need for longitudinal and multimodal approaches to elucidate developmental pathways and guide personalized, mechanism-based prevention and intervention strategies.

## Figures and Tables

**Table 1:** Clinical and demographic characteristics of the sample divided by genotype. CTQ, Childhood Trauma Questionnaire.

	T/T Homozygotes (*n* = 76)	*C Carriers (*n* = 61)	*t*/*χ*^2^	*p*-Value
Age	47.76 ± 11.24	48.43 ± 12.18	−0.330	0.742
Sex (M/F)	27/49	25/36	0.428	0.513
Age at onset of the illness	30.68 ± 11.23	28.79 ± 11.64	0.967	0.335
Total number of illness episodes	8.33 ± 8.47	10.66 ± 10.83	−1.374	0.172
Education (yrs at school)	12.66 ± 3.71	11.79 ± 4.05	1.311	0.871
Lithium treatment in the last six mounths (yes/no)	29/47	29/32	1.220	0.269
CTQ total score	40.24 ± 11.63	37.20 ± 8.73	1.747	0.083
Physical abuse	6.49 ± 3.11	5.92 ± 1.39	1.428	0.156
Emotional abuse	9.11 ± 4.62	7.49 ± 3.04	2.453	0.015 *
Sexual abuse	5.46 ± 1.41	5.33 ± 0.98	0.624	0.534
Physical neglet	6.66 ± 2.26	6.64 ± 2.41	0.046	0.963
Emotional neglet	12.33 ± 4.59	12.11 ± 5.06	0.259	0.796
Minimization	8.96 ± 2.52	9.31 ± 3.12	−0.703	0.483

Note: Statistical significance was set at *p* < 0.05; asterisks indicate statistically significant *p*-values. *t* was used for continuous variables, and *χ*^2^ for categorical variables.

**Table 2:** Clinical and demographic characteristics of the sample divided by sex. CTQ, Childhood Trauma Questionnaire.

	Females (*n* = 85)	Males (*n* = 52)	*t/χ* ^2^	*p*-Value
Age	48.73 ± 10.75	46.96 ± 12.98	0.862	0.390
Age at onset of the illness	29.54 ± 11.05	30.33 ± 12.07	−0.390	0.697
Total number of illness episodes	9.36 ± 9.58	9.37 ± 9.80	0.000	1.000
Education (yrs at school)	11.78 ± 4.04	13.00 ± 3.64	−1.812	0.073
Lithium treatment in the last six mounths (yes/no)	36/49	22/30	0.000	0.996
CTQ total score	39.60 ± 11.39	37.71 ± 8.89	1.082	0.281
Physical abuse	6.20 ± 2.31	6.29 ± 2.80	−0.200	0.842
Emotional abuse	8.87 ± 4.34	7.60 ± 3.46	1.896	0.060
Sexual abuse	5.55 ± 1.49	5.14 ± 0.57	2.223	0.028 *
Physical neglet	6.84 ± 2.48	6.35 ± 2.02	1.200	0.232
Emotional neglet	12.02 ± 4.94	12.58 ± 4.55	−0.655	0.513
Minimization	8.94 ± 2.78	9.40 ± 2.91	−0.929	0.354

Note: Statistical significance was set at *p* < 0.05; asterisks indicate statistically significant *p*-values. *t* was used for continuous variables, and *χ*^2^ for categorical variables.

**Table 3:** WM regions of significant effect of physical abuse on FA and RD. Dimensions of the clusters (number of voxels), localization of signal peaks (MNI coordinates and peak) and associated white matter tracts are reported for regions showing significant within-group (*C carriers and T/T homozygotes) associations between physical abuse and TBSS measures.

DTI Measures	Numbers of Voxels	MNI Coordinates (x,y,z) Peak	WM Tracts Included in the Cluster
Fractional Anisotropy (FA) *C carriers	52084	9, 4, 26 Body of corpus callosum	- Genu of corpus callosum - Body of corpus callosum - Splenium of corpus callosum - Anterior limb of internal capsule (L/R) - Posterior limb of internal capsule (L/R) - Anterior corona radiata (L/R) - Superior corona radiata (L/R) - Posterior corona radiata (L/R) - Posterior thalamic radiation (L/R) - Superior longitudinal fasciculum (R)
Fractional Anisotropy (FA) T/T homozygotes	33797	−29, −30, 8 Retrolenticular part of internal capsule (left)	- Body of corpus callosum - Splenium of corpus callosum - Anterior limb of internal capsule (L/R) - Posterior limb of internal capsule (L/R) - Retrolenticular part of internal capsule (L/R) - Anterior corona radiata (L/R) - Superior corona radiata (L/R) - Posterior corona radiata (L/R) - Posterior thalamic radiation (L/R) - Superior longitudinal fasciculum (L/R) - External capsule L
Radial Diffusivity (RD) *C carriers	25539	1, 9, 22 Body of corpus callosum	- Genu of corpus callosum - Body of corpus callosum - Splenium of corpus callosum - Anterior corona radiata (L/R) - Superior corona radiata (L/R) - Posterior corona radiata (L/R) - Superior longitudinal fasciculum
Radial Diffusivity (RD) T/T homozygotes	2966 530	−15, −32, 29 Splenium of corpus callosum 14, −38, 24 Splenium of corpus callosum	- Body of corpus callosum - Splenium of corpus callosum - Posterior limb of internal capsule (L) - Anterior corona radiata (L) - Superior corona radiata (L) - Posterior corona radiata (L) - Posterior thalamic radiation (L) - Superior longitudinal fasciculum (L) - Posterior thalamic radiation (L) - Retrolenticular part of internal capsule (L) - External capsule L - Body of corpus callosum - Splenium of corpus callosum - Posterior corona radiata (R) - Cingulum (R)

**Table 4:** WM regions of significant effect of physical abuse on FA and RD. Dimensions of the clusters (number of voxels), localization of signal peaks (MNI coordinates and peak) and associated white matter tracts are reported for regions showing significant within-group (females *C carriers and males T/T homozygotes) associations between physical abuse and TBSS measures.

DTI Measures	Numbers of Voxels	MNI Coordinates (x,y,z) Peak	WM Tracts Included in the Cluster
Fractional Anisotropy (FA) Females *C carriers	35196	8, 2, 26 Body of corpus callosum	- Genu of corpus callosum - Body of corpus callosum - Splenium of corpus callosum - Anterior limb of internal capsule (L/R) - Posterior limb of internal capsule (L/R) - Anterior corona radiata (L/R) - Superior corona radiata (L/R) - Posterior corona radiata (L/R) - Posterior thalamic radiation (L/R)
Fractional Anisotropy (FA) Males T/T homozygotes	20665	13, −37, 23 Splenium of corpus callosum	- Body of corpus callosum - Splenium of corpus callosum - Anterior limb of internal capsule (L/R) - Posterior limb of internal capsule (L/R) - Retrolenticular part of internal capsule (L/R) - Anterior corona radiata (L/R) - Superior corona radiata (L/R) - Posterior corona radiata (L/R) - Posterior thalamic radiation (R) - Superior longitudinal fasciculum (R) - External capsule L
Radial Diffusivity (RD) Females *C carriers	10100	2, 9, 22 Body of corpus callosum	- Genu of corpus callosum - Body of corpus callosum - Splenium of corpus callosum - Anterior corona radiata (L/R) - Superior corona radiata (L/R) - Posterior corona radiata (R)
Radial Diffusivity (RD) Males T/T homozygotes	20308	−27, 11, 14 External Capsule (left)	- Genu of corpus callosum - Body of corpus callosum - Splenium of corpus callosum - Anterior limb of internal capsule (L/R) - Posterior limb of internal capsule (L/R) - Anterior corona radiata (L) - Superior corona radiata (L) - Posterior corona radiata (L) - Posterior thalamic radiation (L) - Superior longitudinal fasciculum (L/R) - External capsule L

**Table 5:** Results of GLZ model with separate slopes on FA. Statistical significance reported (*p*-value); FA, fractional anisotropy.

FA	Estimate	Wald Stat.	Lower CL 95%	Upper CL 95%	*p*-Value
SEX (female) x CLOCK T/T x Physical Abuse	−0.002801	1.6008	−0.0071	0.0011	0.2057
SEX (female) x CLOCK*C x Physical Abuse	−0.010389	6.3288	−0.0184	−0.0022	0.0118 *
SEX (male) x CLOCK T/T x Physical Abuse	−0.004443	4.0392	−0.0087	−0.0001	0.0444 *
SEX (male) x CLOCK*C x Physical Abuse	−0.012656	6.2201	−0.0226	−0.0027	0.0126 *

Note: Statistical significance was set at *p* < 0.05; asterisks indicate statistically significant *p*-values.

**Table 6:** Results of GLZ model with separate slopes on RD. Statistical significance reported (*p*-value); RD, radial diffusivity.

RD	Estimate	Wald Stat.	Lower CL 95%	Upper CL 95%	*p*-Value
SEX (female) x CLOCK T/T x Physical Abuse	0.000003	0.9378	−0.000003	0.000008	0.3328
SEX (female) x CLOCK*C x Physical Abuse	0.000012	5.5610	−0.000002	0.000022	0.0183 *
SEX (male) x CLOCK T/T x Physical Abuse	0.000007	6.0911	−0.000001	0.000012	0.0135 *
SEX (male) x CLOCK*C x Physical Abuse	0.000018	8.2867	−0.000006	0.000031	0.0039 *

Note: Statistical significance was set at *p* < 0.05; asterisks indicate statistically significant *p*-values.

## Data Availability

The original contributions presented in this study are included in the article. Further inquiries can be directed to the corresponding authors.

## Supplementary Materials

The following supporting information can be downloaded at: https://www.mdpi.com/article/10.3390/genes17080912/s1. Figure S1(a): interaction between CTQ Emotional Neglet and CLOCK 3111T/C on FA in the whole sample; Figure S1(b): interaction between CTQ Emotional Neglet and CLOCK 3111T/C on RD in the whole sample; Figure S1(c): interaction between CTQ Emotional Neglet and CLOCK 3111T/C on FA in females; Figure S1(d): interaction between CTQ Emotional Neglet and CLOCK 3111T/C on RD in females; Figure S1(e): interaction between CTQ Emotional Neglet and CLOCK 3111T/C on FA in males; Figure S1(f): interaction between CTQ Emotional Neglet and CLOCK 3111T/C on RD in males; Figure S2(a): interaction between CTQ Physical Neglet and CLOCK 3111T/C on FA in the whole sample; Figure S2(b): interaction between CTQ Physical Neglet and CLOCK 3111T/C on RD in the whole sample; Figure S2(c): interaction between CTQ Physical Neglet and CLOCK 3111T/C on FA in females; Figure S2(d): interaction between CTQ Physical Neglet and CLOCK 3111T/C on RD in females; Figure S2(e): interaction between CTQ Physical Neglet and CLOCK 3111T/C on FA in males; Figure S2(f): interaction between CTQ Physical Neglet and CLOCK 3111T/C on RD in males; Table S1: Clinical and demographic characteristics of patients with BD and healthy controls; Supplementary Results: Voxel-wise ANOVA across groups (*C carriers, TT homozygotes, healthy controls); Figure S3(a): Voxel-wise post hoc pairwise contrast of FA (HC vs *C carriers), derived from a one-way ANOVA (TFCE-corrected, *p* < 0.05). White matter tracts where CLOCK*C carriers show lower FA than healthy controls. The color bar represents 1− *p* values for the observed differences. Coordinates are reported as z-values in standard Montreal Neurological Institute (MNI) space.; Figure S3(b): Voxel-wise post hoc pairwise contrast of AD (HC vs *C carriers), derived from a one-way ANOVA (TFCE-corrected, *p* < 0.05). White matter tracts where CLOCK*C carriers show higher AD than healthy controls. The color bar represents 1− *p* values for the observed differences. Coordinates are reported as z-values in standard Montreal Neurological Institute (MNI) space.; Figure S3(c): Voxel-wise post hoc pairwise contrast of RD (HC vs *C carriers), derived from a one-way ANOVA (TFCE-corrected, *p* < 0.05). White matter tracts where CLOCK*C carriers show higher RD than healthy controls. The color bar represents 1− *p* values for the observed differences. Coordinates are reported as z-values in standard Montreal Neurological Institute (MNI) space.; Figure S3(d): Voxel-wise post hoc pairwise contrast of MD (HC vs C carriers), derived from a one-way ANOVA (TFCE-corrected, *p* < 0.05). White matter tracts where CLOCK*C carriers show higher MD than healthy controls. The color bar represents 1− *p* values for the observed differences. Coordinates are reported as z-values in standard Montreal Neurological Institute (MNI) space. Figure S4(a): Voxel-wise comparison of AD between *C carriers and TT homozygotes using two-sample *t*-tests (TFCE-corrected, *p* < 0.05). White matter tracts where CLOCK*C carriers show higher AD than CLOCK T/T homozygotes. The color bar refers to 1− *p* values for the observed differences. Numbers are z coordinates in the standard Montreal Neurological Institute (MNI) space. Figure S4(b): Voxel-wise comparison of RD between *C carriers and TT homozygotes using two-sample *t*-tests (TFCE-corrected, *p* < 0.05). White matter tracts where CLOCK*C carriers show higher RD than CLOCK T/T homozygotes. The color bar refers to 1− *p* values for the observed differences. Numbers are z coordinates in the standard Montreal Neurological Institute (MNI) space. Figure S4(c): Voxel-wise comparison of MD between *C carriers and TT homozygotes using two-sample *t*-tests (TFCE-corrected, *p* < 0.05). White matter tracts where CLOCK*C carriers show higher MD than CLOCK T/T homozygotes. The color bar refers to 1− *p* values for the observed differences. Numbers are z coordinates in the standard Montreal Neurological Institute (MNI) space; Figure S5(a) and S5(b): 3D visualization of significant effect of physical abuse on FA respectively in right and left hemispheres in CLOCK*C carriers; Figure S5(c) and S5(d): 3D visualization of significant effect of physical abuse on RD respectively in right and left hemispheres in CLOCK*C carriers; Figure S6(a) and S6(b): 3D visualization of significant effect of physical abuse on FA respectively in right and left hemispheres in T/T homozygotes; Figure S6(c) and S6(d): 3D visualization of significant effect of physical abuse on RD respectively in right and left hemispheres in T/T homozygotes; Figure S7(a) and S7(b): 3D visualization of significant effect of physical abuse on FA respectively in right and left hemispheres in females CLOCK*C carriers; Figure S7(c) and S7(d): 3D visualization of significant effect of physical abuse on RD respectively in right and left hemispheres in females CLOCK*C carriers; Figure S8(a) and S8(b): 3D visualization of significant effect of physical abuse on FA respectively in right and left hemispheres in males T/T homozygotes; Figure S8(c) and S8(d): 3D visualization of significant effect of physical abuse on RD respectively in right and left hemispheres in males T/T homozygotes.

## Abbreviations

The following abbreviations are used in this manuscript:

BD	Bipolar Disorder
ACES	Adverse Childhood Experiences
WM	White Matter
FA	Fractional Anisotropy
RD	Radial Diffusivity
AD	Axial Diffusivity
MD	Mean Diffusivity
DTI	Diffusion Tensor Imaging
SNC	Suprachiasmatic Nucleus
CTQ	Childhood Trauma Questionnaire
