# Supplementary material for: CLOCK 3111T/C Polymorphism and Sex Moderate the Effect of Childhood Trauma on White Matter Microstructure in Bipolar Disorder
Source: Genes (Basel). 2026 Jul 31;17(8):912. doi: 10.3390/genes17080912 (PMC13511937; doi:10.3390/genes17080912)
Supplement: Supplementary file 1 [file genes-17-00912-s001.zip › genes-4434452-supplementary.pdf]

Supplementary Materials

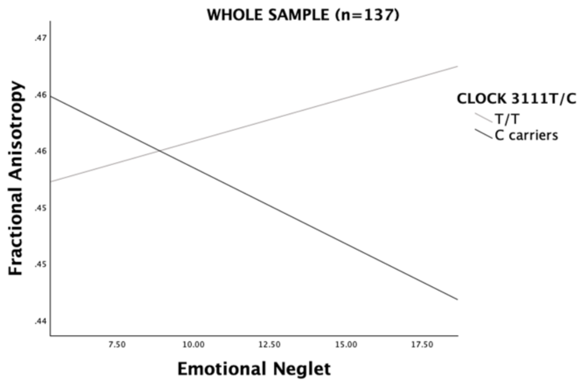

Figure S1(a): interaction between CTQ Emotional Neglet and rs1801260 on FA in the whole sample

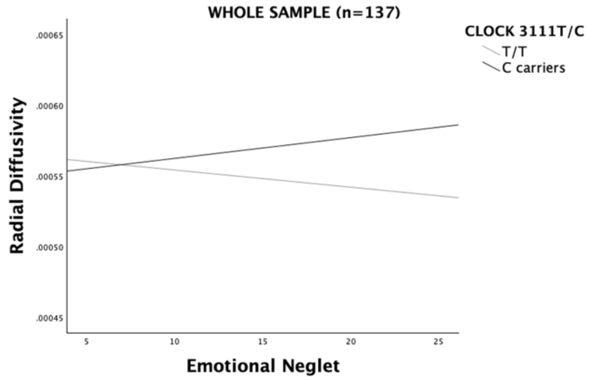

Figure S1(b): interaction between CTQ Emotional Neglet and rs1801260 on RD in the whole sample

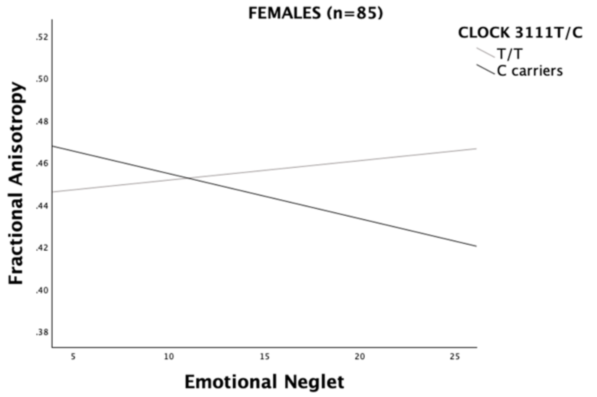

Figure S1(c): interaction between CTQ Emotional Neglet and rs1801260 on FA in females

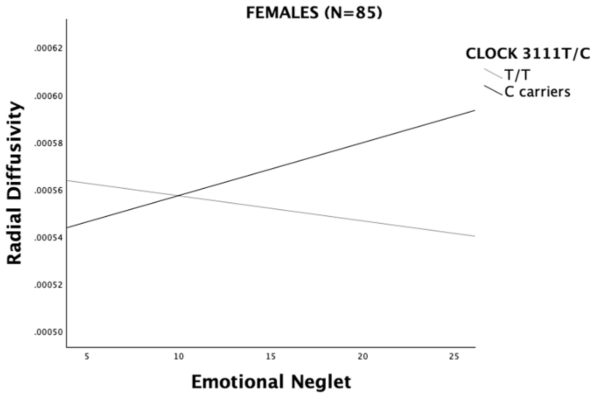

Figure S1(d): interaction between CTQ Emotional Neglet and rs1801260 on RD in females

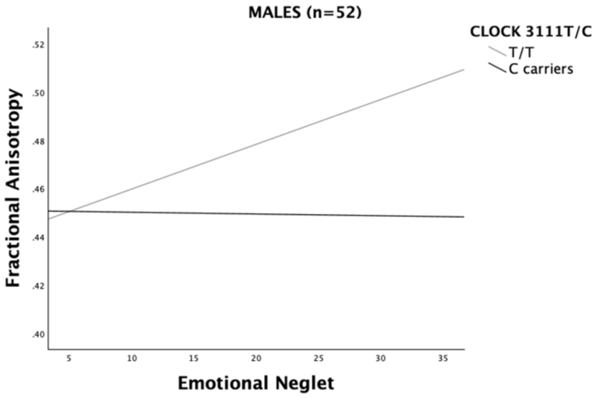

Figure S1(e): interaction between CTQ Emotional Neglet and rs1801260 on FA in males

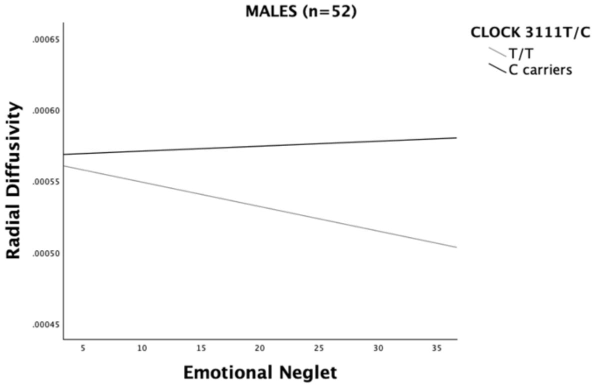

Figure S1(f): interaction between CTQ Emotional Neglet and rs1801260 on RD in males

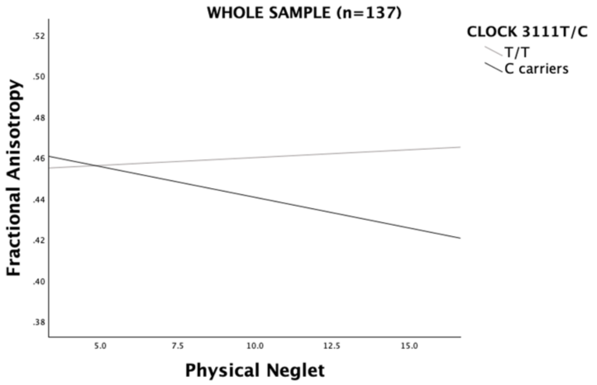

Figure S2(a): interaction between CTQ Physical Neglet and rs1801260 on FA in the whole sample

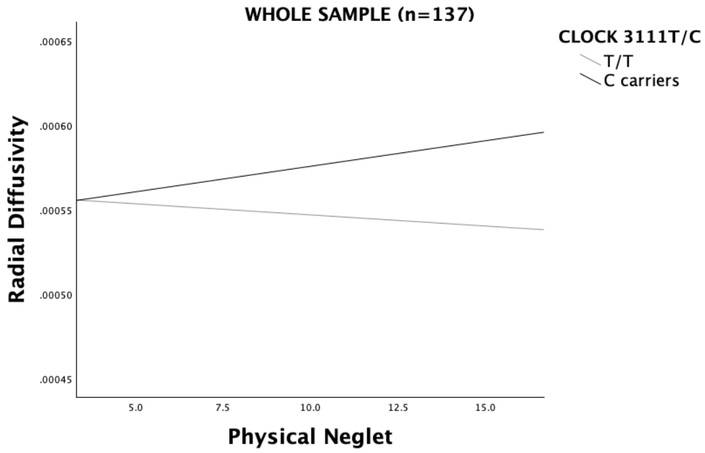

Figure S2(b): interaction between CTQ Physical Neglet and rs1801260 on RD in the whole sample

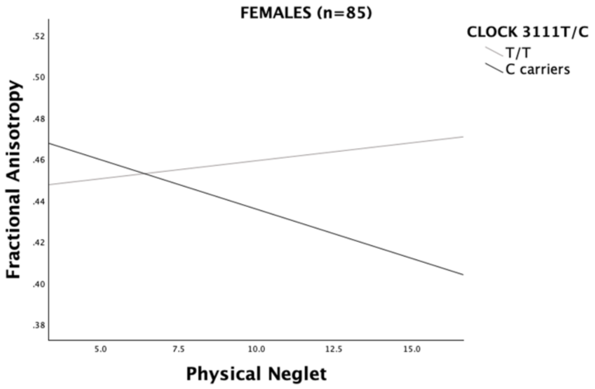

Figure S2(c): interaction between CTQ Physical Neglet and rs1801260 on FA in females

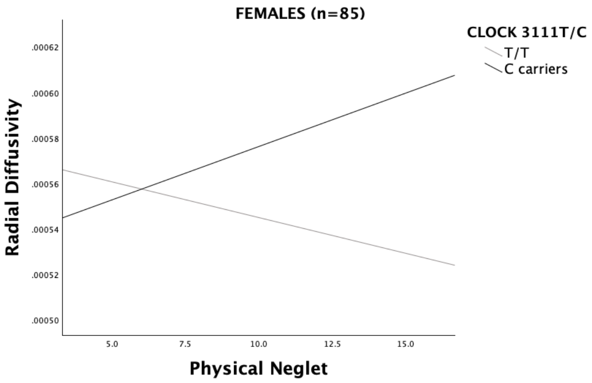

Figure S2(d): interaction between CTQ Physical Neglet and rs1801260 on RD in females

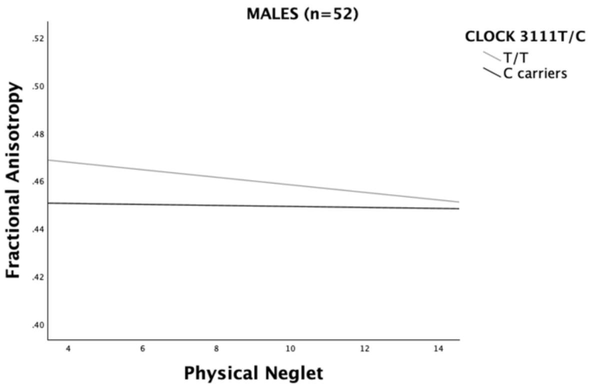

Figure S2(e): interaction between CTQ Physical Neglet and rs1801260 on FA in males

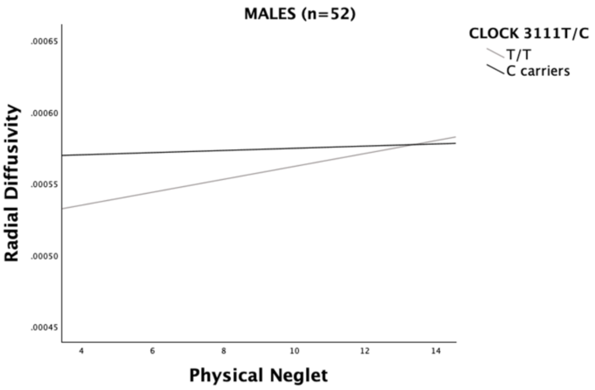

Figure S2(f): interaction between CTQ Physical Neglet and rs1801260 on RD in males

Supplementary Results:

Table S1. Clinical and demographic characteristics of patients with BD and healthy controls

	BD (n=137)	Healthy control (n=107)	t/χ^2	p-value
Age	48.06 ± 11.635	45.57 ± 11.066	1.693	0.092
Sex (M/F)	52/85	53/54	3.284	0.070
Education (yrs at school)	12.27 ± 3.875	14.25 ± 3.734	-4.028	**0.001***

Note: Statistical significance was set at $p < 0.05$; asterisks indicate statistically significant p-values. t was used for continuous variables, and χ^2 for categorical variables.

Clinical and demographic characteristics
Tables S1 summarizes the clinical and demographic characteristics of the sample, which included 137 patients with bipolar disorder (BD) and 107 healthy controls (HC). No significant between-group differences were observed in demographic variables, except for education, which was significantly higher in HC compared to BD patients (HC: 14.25 ± 3.73; BD: 12.27 ± 3.88; t (242)= -4.028, p=.001).

Voxel-wise ANOVA across groups (C carriers, TT homozygotes, healthy controls)
Voxel-wise one-way ANOVAs were conducted in FSL (Randomise), using TFCE correction and adjusting for sex, age, and scan as covariates. Group differences were assessed in diffusion metrics (FA, MD, RD, and AD) among C carriers, TT homozygotes, and healthy controls. A significant main effect of group was found for all diffusion metrics, including fractional anisotropy (FA: $p = 0.0106$), radial diffusivity (RD: $p = 0.0012$), axial diffusivity (AD: $p < 0.001$), and mean diffusivity (MD: $p = 0.002$). Post hoc analyses showed that, compared with C carriers, healthy controls exhibited significantly higher FA ($p = 0.002$) (Figure S3a) and significantly lower AD ($p = 0.021$) (Figure S3b), RD ($p < 0.001$) (Figure S3c), and MD ($p = 0.001$) (Figure S3d). These effects were localized in the corpus callosum, corona radiata, posterior thalamic radiation, external capsule, and superior longitudinal fasciculus.
No significant differences were observed between TT homozygotes and healthy controls.

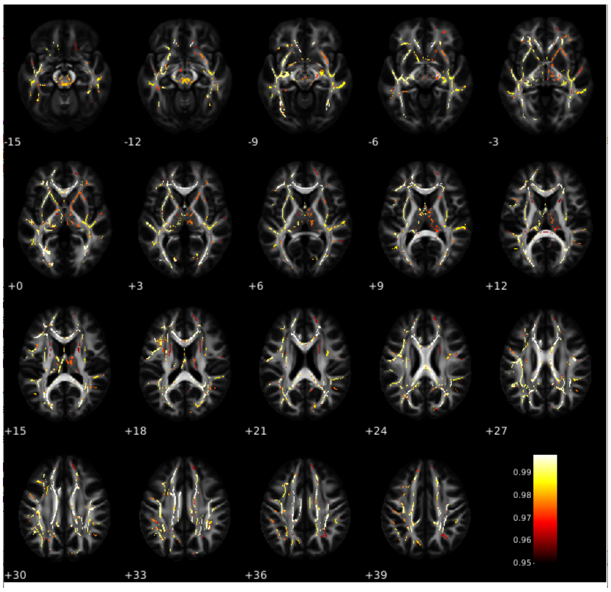

Figure S3(a): Voxel-wise post hoc pairwise contrast of FA (HC vs *C carriers), derived from a one-way ANOVA (TFCE-corrected, $p < .05$). White matter tracts where CLOCK*C carriers show lower FA than healthy controls. The color bar represents 1−p values for the observed differences. Coordinates are reported as z-values in standard Montreal Neurological Institute (MNI) space.

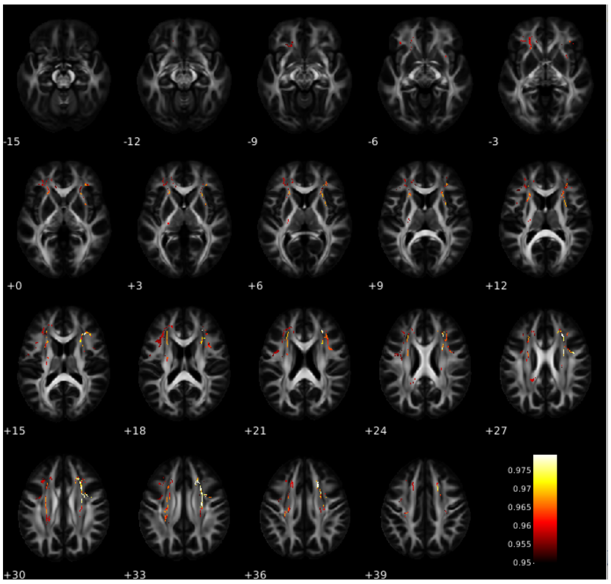

Figure S3(b): Voxel-wise post hoc pairwise contrast of AD (HC vs *C carriers), derived from a one-way ANOVA (TFCE-corrected, $p < .05$). White matter tracts where CLOCK*C carriers show higher AD than healthy controls. The color bar represents 1−p values for the observed differences. Coordinates are reported as z-values in standard Montreal Neurological Institute (MNI) space.

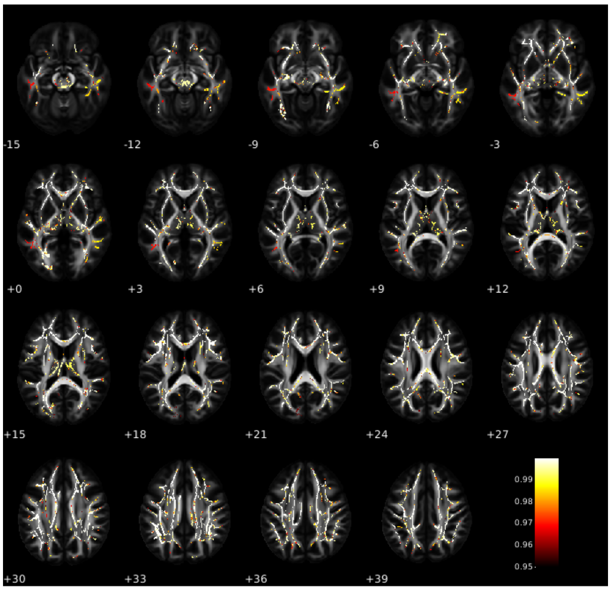

Figure S3(c): Voxel-wise post hoc pairwise contrast of RD (HC vs *C carriers), derived from a one-way ANOVA (TFCE-corrected, $p < .05$). White matter tracts where CLOCK*C carriers show higher RD than healthy controls. The color bar represents $1-p$ values for the observed differences. Coordinates are reported as z-values in standard Montreal Neurological Institute (MNI) space.

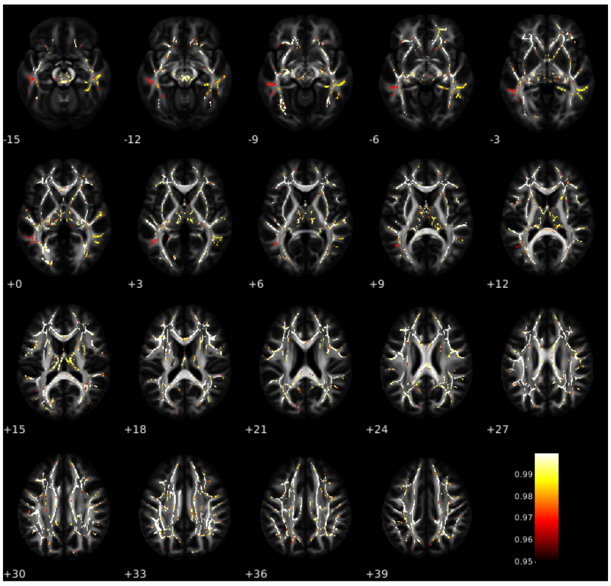

Figure S3(d): Voxel-wise post hoc pairwise contrast of MD (HC vs C carriers), derived from a one-way ANOVA (TFCE-corrected, $p < .05$). White matter tracts where CLOCK*C carriers show higher MD than healthy controls. The color bar represents $1-p$ values for the observed differences. Coordinates are reported as z-values in standard Montreal Neurological Institute (MNI) space.

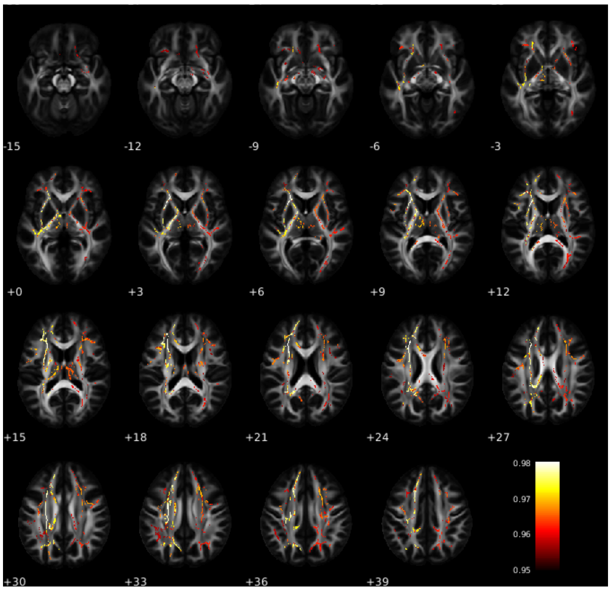

Figure S4(a): Voxel-wise comparison of AD between *C carriers and TT homozygotes using two-sample t-tests (TFCE-corrected, $p < .05$). White matter tracts where CLOCK*C carriers show higher AD than CLOCK T/T homozygotes. The color bar refers to $1-p$ values for the observed differences. Numbers are z coordinates in the standard Montreal Neurological Institute (MNI) space.

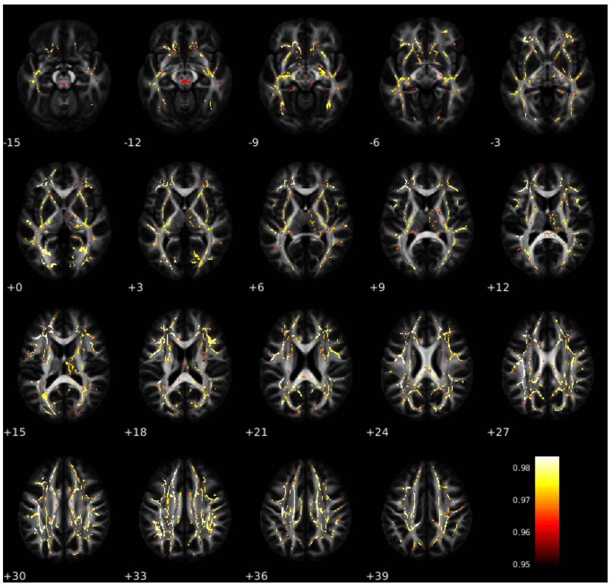

Figure S4(b): Voxel-wise comparison of RD between *C carriers and TT homozygotes using two-sample t-tests (TFCE-corrected, $p < .05$). White matter tracts where CLOCK*C carriers show higher RD than CLOCK T/T homozygotes. The color bar refers to $1-p$ values for the observed differences. Numbers are z coordinates in the standard Montreal Neurological Institute (MNI) space.

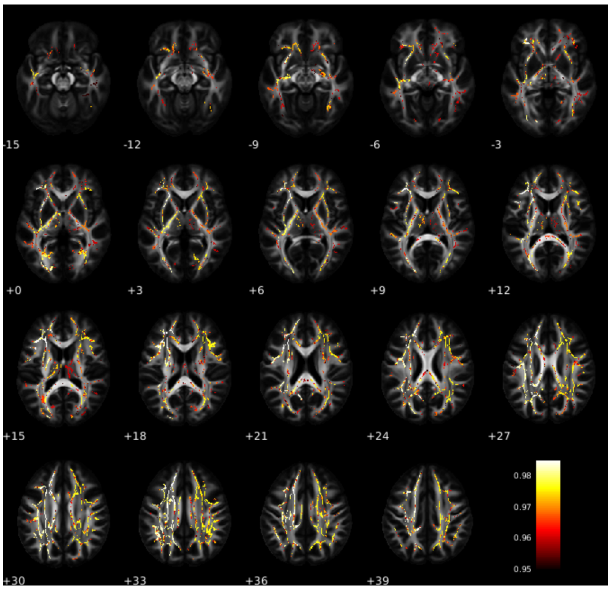

Figure S4(c): Voxel-wise comparison of MD between *C carriers and TT homozygotes using two-sample t-tests (TFCE-corrected, $p < .05$). White matter tracts where CLOCK*C carriers show higher MD than CLOCK T/T homozygotes. The color bar refers to 1–p values for the observed differences. Numbers are z coordinates in the standard Montreal Neurological Institute (MNI) space.

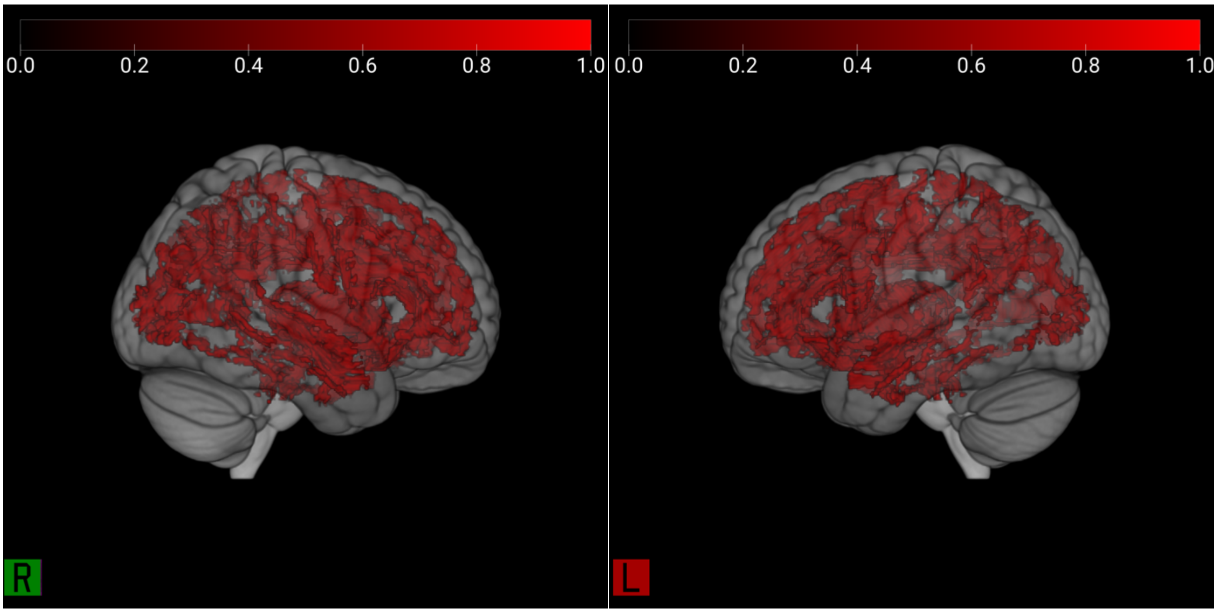

Figure S5(a) and S5(b): 3D visualization of significant effect of physical abuse on FA respectively in right and left hemispheres in CLOCK*C carriers.

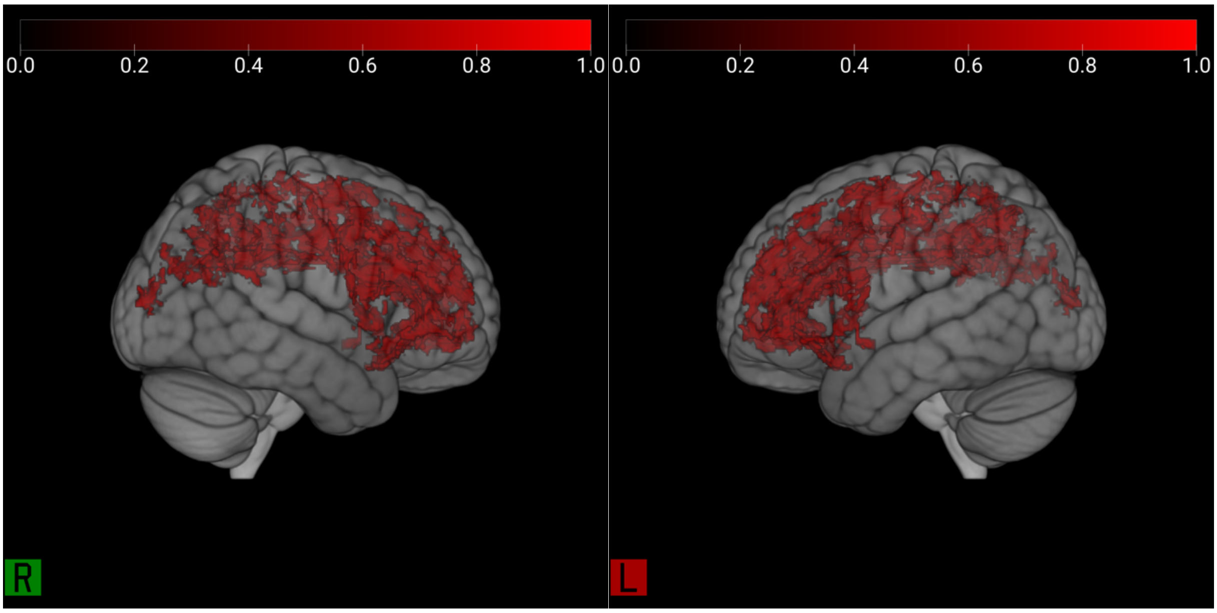

Figure S5(c) and S5(d): 3D visualization of significant effect of physical abuse on RD respectively in right and left hemispheres in CLOCK*C carriers

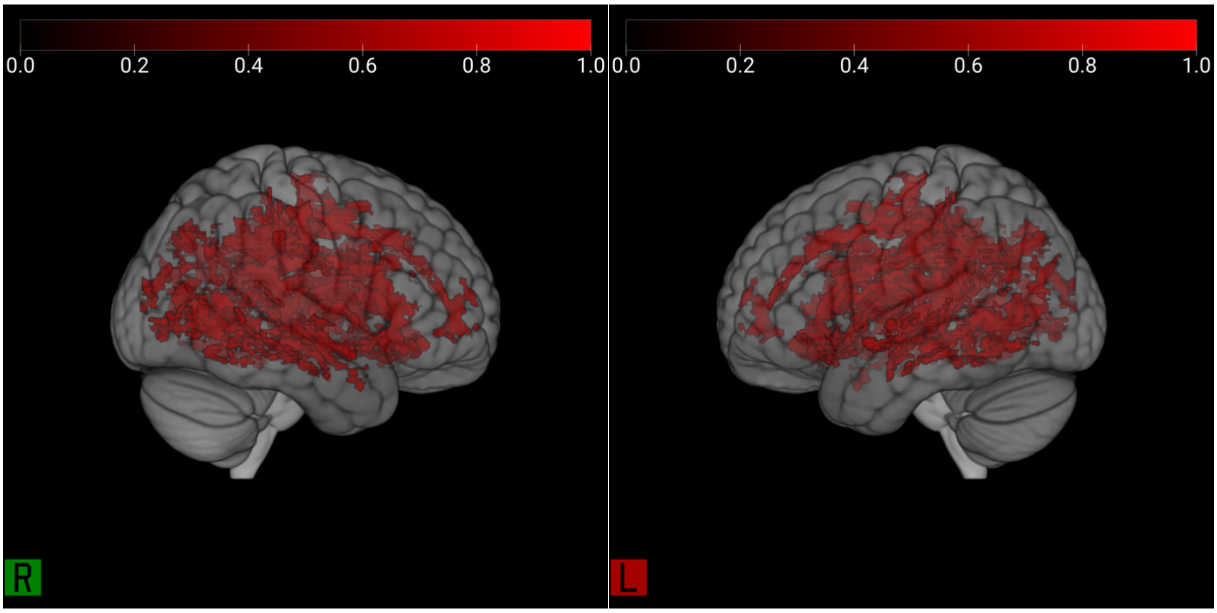

Figure S6(a) and S6(b): 3D visualization of significant effect of physical abuse on FA respectively in right and left hemispheres in T/T homozygotes

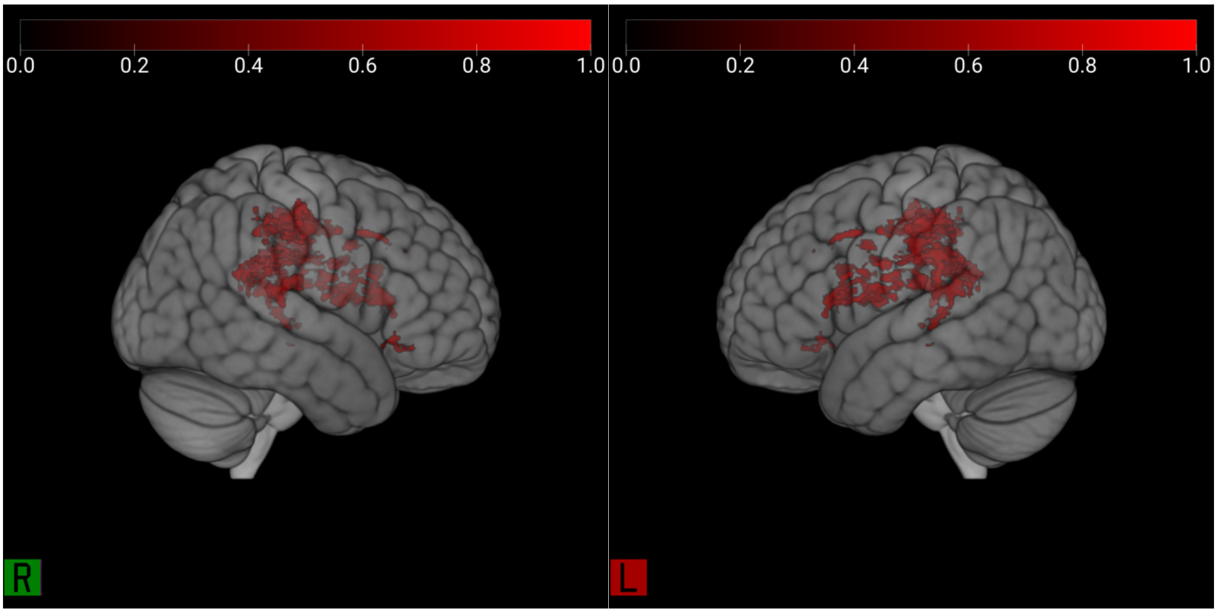

Figure S6(c) and S6(d): 3D visualization of significant effect of physical abuse on RD respectively in right and left hemispheres in T/T homozygotes

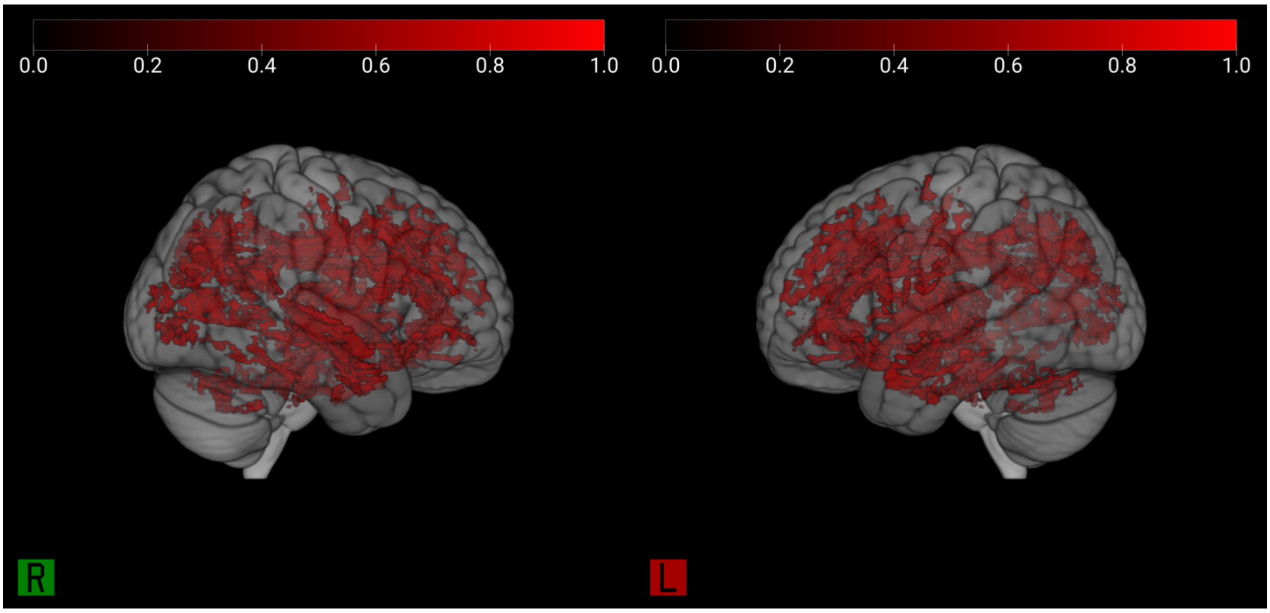

Figure S7(a) and S7(b): 3D visualization of significant effect of physical abuse on FA respectively in right and left hemispheres in females CLOCK*C carriers

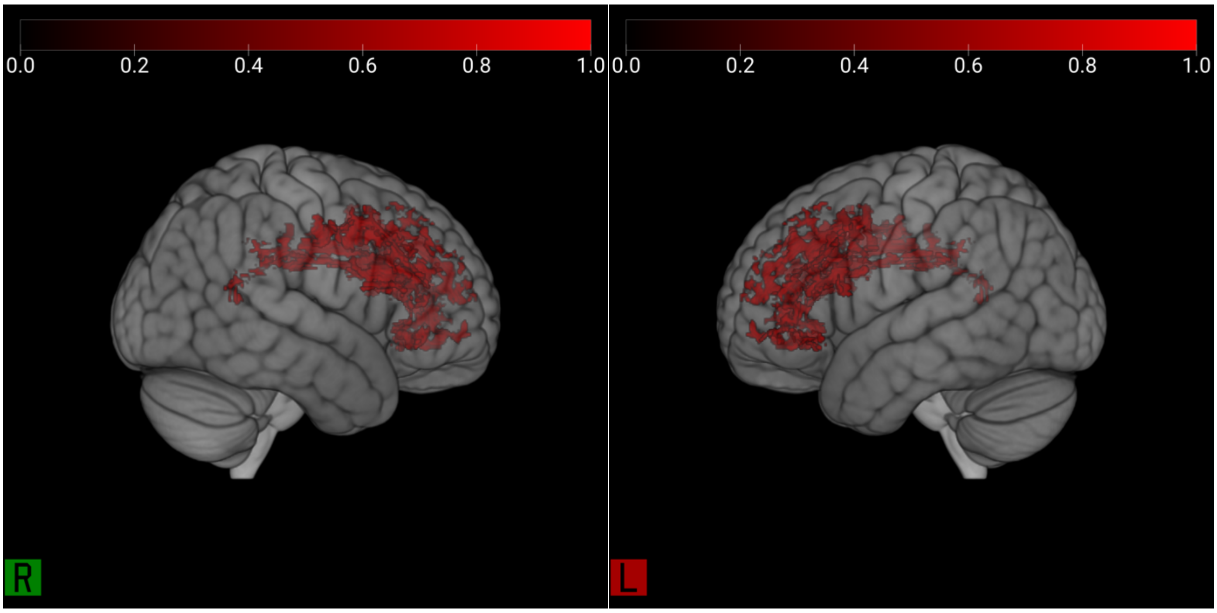

Figure S7(c) and S7(d): 3D visualization of significant effect of physical abuse on RD respectively in right and left hemispheres in females CLOCK*C carriers

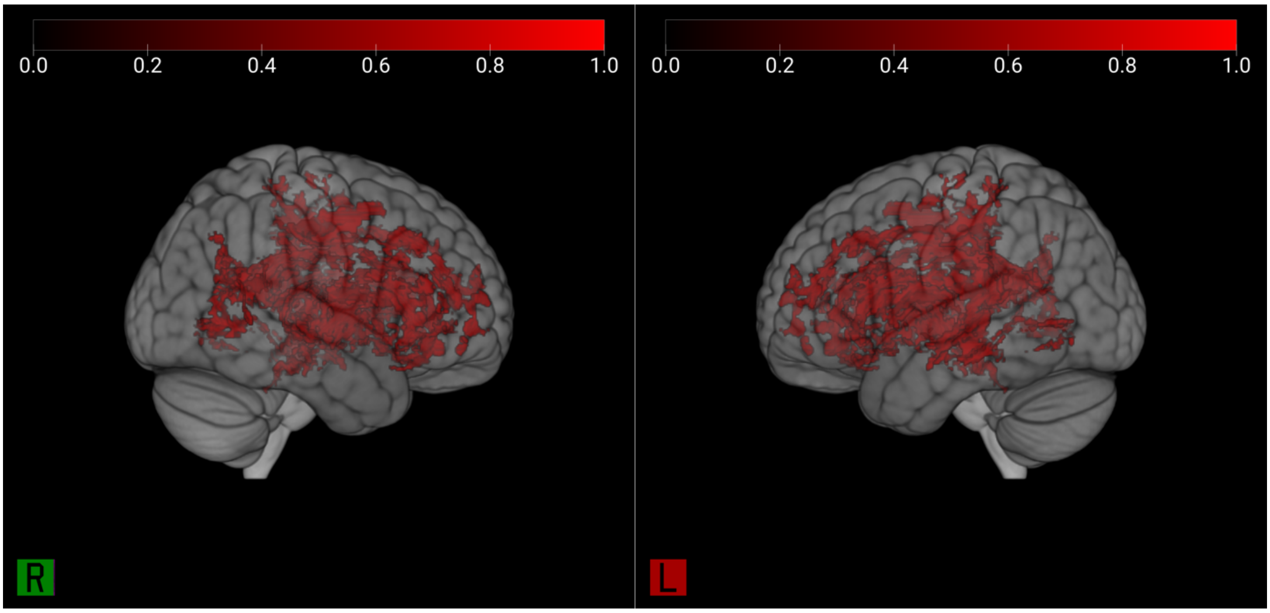

Figure S8(a) and S8(b): 3D visualization of significant effect of physical abuse on FA respectively in right and left hemispheres in males T/T homozygotes

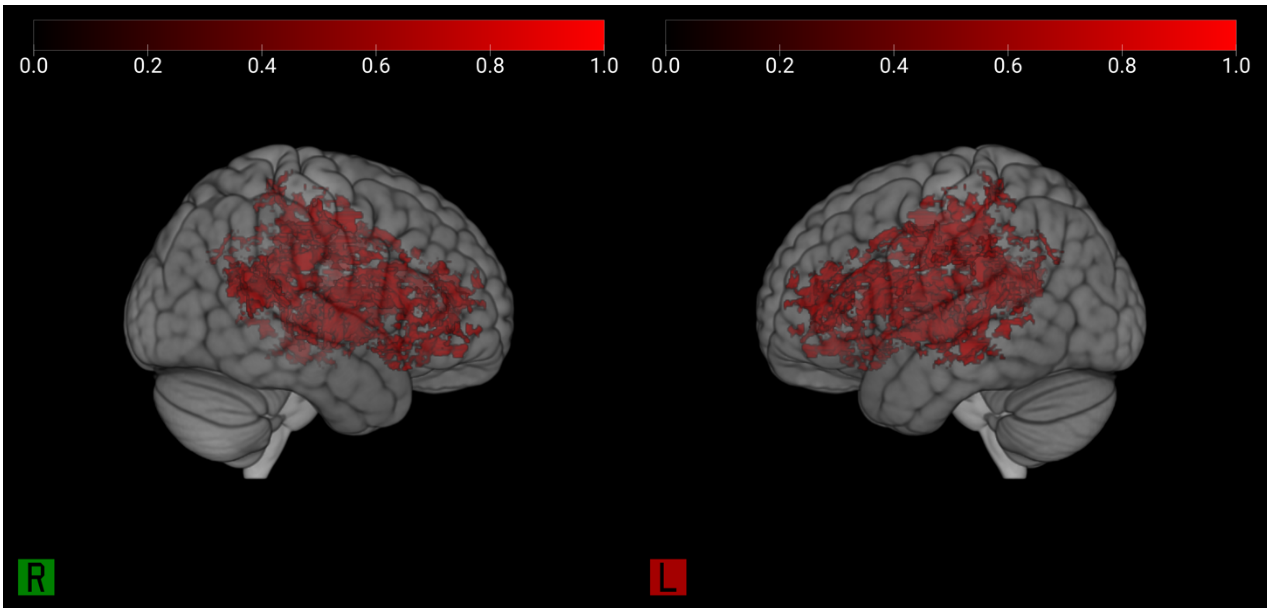

Figure S8(c) and S8(d): 3D visualization of significant effect of physical abuse on RD respectively in right and left hemispheres in males T/T homozygotes
